# Bridging Links between Long Noncoding RNA HOTAIR and HPV Oncoprotein E7 in Cervical Cancer Pathogenesis

**DOI:** 10.1038/srep11724

**Published:** 2015-07-08

**Authors:** Sweta Sharma, Paramita Mandal, Tamal Sadhukhan, Rahul Roy Chowdhury, Nidhu Ranjan Mondal, Biman Chakravarty, Tanmay Chatterjee, Sudipta Roy, Sharmila Sengupta

**Affiliations:** 1 National Institute of Biomedical Genomics, Netaji Subhas Sanatorium, 2^nd^ Floor, P.O. N.S.S, Kalyani 741251, West Bengal, India; 2 Department of Gynecology, Saroj Gupta Cancer Centre and Research Institute, Kolkata,India; 3 Sri Aurobindo Seva Kendra, 1H, Gariahat Road (S) Jodhpur Park, Kolkata-700068, West Bengal, India

## Abstract

Human Papillomavirus (HPV) type 16 oncoprotein E7 plays a major role in cervical carcinogenesis by interacting with and functionally inactivating various host regulatory molecules. Long noncoding RNA (lncRNA) HOTAIR is one such regulator that recruits chromatin remodelling complex PRC2, creating gene silencing H3K27 me3 marks. Hence, we hypothesized that HOTAIR could be a potential target of E7, in HPV16 related cervical cancers (CaCx). We identified significant linear trend of progressive HOTAIR down-regulation through HPV negative controls, HPV16 positive non-malignants and CaCx samples. Majority of CaCx cases portrayed HOTAIR down-regulation in comparison to HPV negative controls, with corresponding up-regulation of HOTAIR target, HOXD10, and enrichment of cancer related pathways. However, a small subset had significantly higher HOTAIR expression, concomitant with high E7 expression and enrichment of metastatic pathways. Expression of HOTAIR and PRC2-complex members (EZH2 and SUZ12), showed significant positive correlation with E7 expression in CaCx cases and E7 transfected C33A cell line, suggestive of interplay between E7 and HOTAIR. Functional inactivation of HOTAIR by direct interaction with E7 could also be predicted by *in silico* analysis and confirmed by RNA-Immunoprecipitation. Our study depicts one of the causal mechanisms of cervical carcinogenesis by HPV16 E7, through modulation of HOTAIR expression and function.

Cervical carcinoma (CaCx) is the second most prevalent cancer among women in India after breast cancer and the fourth most prevalent cancer among women worldwide^1^,^2^. Human Papillomavirus (HPV) is considered as the major etiologic contributor to the development of CaCx and is found in 99.7% of all the cases, of which, the high-risk types HPV16 and 18 are the most prevalent ones^3^,^4^. HPV16 alone contributes to more than 50% of the CaCx cases globally^5^.

HPV16 acts by frequently integrating into the host chromosome and replicates along with the host genome, which results in E2 gene disruption and consistent expression of the two HPV oncoproteins E6 and E7 due to loss of E2 repressor activity^6^. The infected epithelial basal cells differentiate from the basal membrane to the superficial zone and the virus particles are shed with the sloughed-off epithelial cells. Moreover, E6 and E7 also facilitate persistence of episomal HPV genomes in undifferentiated cells of the cervical epithelium^7^. It is well established that oncoproteins E6 and E7 are the major transforming agents, leading to carcinogenesis. While E6 regulates the decay of the tumor suppressor p53, E7 leads to cellular transformation by interacting with the PDZ domain of cellular proteins and pRb, thereby contributing to neoplastic progression^8^.

However, recent proteomics based studies^9^,^10^ have revealed other potential transforming targets of viral oncoproteins E6 and E7 and hence, novel mechanisms leading to cervical carcinogenesis. Such studies have identified that viral oncoproteins are known to interact with multitudes of host encoded molecules. This includes transcriptional regulators like Polycomb repressive complex containing E2F6^11^,^12^. Interestingly, these HPV16 E7 interacting E2F6 transcription factors possess the ability of transcriptionally regulating EZH2, a member of the Polycomb Repressive Complex 2 (PRC2)^13^,^14^. The PRC2 complex, on the other hand, has been reported to interact with a large number of long noncoding RNAs (lncRNAs). These lncRNAs have emerged as important regulators of transcription and disease progression, particularly of various cancers^15^,^16^. They are more than 200 nucleotides in length, do not code for any protein^17^, and possess a variety of functions, acting in both *cis* and *trans* fashion. This includes regulation of chromatin modifying complexes, functioning as antisense molecules to protein coding genes, organizing enhancer-activity. They also have strong implications in genomic reprogramming, such as induction of differentiation of somatic cells to pluripotent stem cells^18^,^19^,^20^. Several lncRNAs have been reported to be differentially expressed in a large variety of disease states, including cancer^21^,^22^,^23^,^24^.

One such regulatory lncRNA differentially expressed in a variety of cancers is HOX transcript antisense intergenic RNA (HOTAIR). HOTAIR is a 2.2 kb lncRNA transcribed from the HOXC cluster located in chromosome 12q13.3^25^. It is known to be deregulated in several cancers^26^,^27^,^28^,^29^ and can regulate global gene expression by associating with chromatin remodelling complexes^30^. HOTAIR bridges the Polycomb repressive complex 2 (PRC2) with the Lysine-specific histone demethylase 1 A complex (LSD1), leading to H3K27 me3 and demethylation of H3K4 me3 marks of their target genes, resulting in gene silencing globally. HOXD10 is one such HOTAIR target, which is silenced through this mechanism^21^,^25^,^30^.

Such global change at the transcriptional level is a common event associated with all cancers, including CaCx cases expressing HPV16 oncoprotein E7. Furthermore, HPV16 positive CaCx cases also portray a characteristic E7-dependent global reduction in the chromatin repressive H3K27 me3 mark^14^,^31^,^32^, indicative of probable impairment of HOTAIR function. Based on this rationale, we hypothesized that one of the mechanisms by which HPV16 E7 mediates its causal effect of activation of cancer-related pathway genes in CaCx pathogenesis, is through interplay with lncRNA HOTAIR. This ultimately affects the function of PRC2-complex recruitment by HOTAIR, which otherwise would normally act by creating chromatin repressive H3K27 me3 marks. Existing reports highlight the deregulation of the lncRNA HOTAIR in CaCx cases as well, reflecting its association with disease prognosis^33^,^34^, based on the metastatic potential of HOTAIR. However, we undertook the present study to test the hypotheses that (i) HOTAIR deregulation is involved in HPV16 related CaCx pathogenesis and (ii) E7 mediated cervical carcinogenesis is brought about through interplay between E7 and HOTAIR.

Our study revealed statistically significant down-regulation of HOTAIR expression in CaCx cases in comparison to the HPV negative controls. We also identified two categories of CaCx cases expressing low and high levels of HOTAIR, the latter depicting enrichment of processes related to cellular movement and metastasis. Further studies helped us to predict and confirm the physical interaction between HOTAIR and E7 oncoprotein. This helped us to propose that HOTAIR could serve as one of the targets of the HPV16 oncoprotein E7 in the process of cervical carcinogenesis.

## Results

### HOTAIR is deregulated in HPV 16 positive cases as compared to the healthy HPV negative controls

To identify the role of HOTAIR in HPV16 positive CaCx cases, we determined HOTAIR expression in HPV negative controls (n = 37), HPV16 positive non-malignants (n = 28) and HPV16 positive CaCx cases (n = 107). The analyzed samples were all histopathologically characterized and confirmed as HPV negative or HPV16 positive by PCR based methods described earlier^35^. The physical status of the HPV16 genome (episomal or integrated) were determined using APOT cum Taqman assay, as described earlier^36^. The CaCx case group comprised of two categories of samples. These were cases (i) harbouring episomal HPV16 (n = 63) and (ii) those with integrated HPV16 (n = 44).

**Figure 1:**
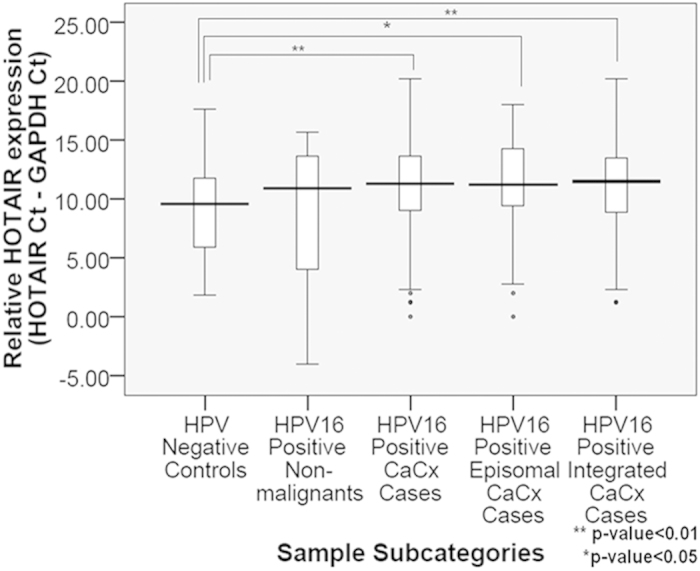
HOTAIR deregulation in cervical cancers. Box plots representing distribution of HOTAIR expression levels among the different categories of cervical samples.

Analysis revealed that HOTAIR was significantly down-regulated by 6.06-fold (p = 0.003) in HPV16 positive CaCx cases, as compared to HPV negative controls (n = 37). HOTAIR deregulation was independent of the physical status of the HPV16 genome, as CaCx cases harbouring both episomal and integrated HPV16 genomes showed comparable levels of HOTAIR down-regulation (episomal: 5.7-fold; p = 0.005 and integrated: 6.7-fold; p = 0.017). HPV16 positive non-malignants also showed HOTAIR down-regulation by 3.24-fold, which was not statistically significant (p = 0.442) (Fig. 1). However, a linear trend of progressive down-regulation of HOTAIR was recorded through HPV negative controls, HPV16 positive non-malignants and HPV16 positive CaCx cases (Supplementary Fig. S1).

### ROC curve analysis to identify CaCx subgroups based on HOTAIR expression

Our observation of HOTAIR down-regulation among CaCx cases, compared to controls, was contrary to HOTAIR up-regulation in a variety of cancer types including hepatocellular carcinoma, breast, colon, oesophageal and colorectal cancers^21^,^26^,^27^,^28^,^29^. Interestingly, however, most of these reports indicated the presence of subgroups among cancers harbouring low and high levels of HOTAIR expression, irrespective of overall up-regulation of HOTAIR expression. Therefore, we undertook further analysis, to identify the existence of any subgroup showing high HOTAIR expression among our CaCx cases.

We used ROC curve analysis to confidently predict the cut-off level of HOTAIR expression, to classify the CaCx cases as expressing high HOTAIR or low HOTAIR. HPV16 positive CaCx cases could thus be subdivided into low and high HOTAIR (LH and HH) groups at a ΔCt cut-off value of 7.79 (Fold change = 2.3 fold; Sensitivity = 0.879 and 1-Specificity = 0.595; p = 0.012), corresponding to an AUC of 0.638 (Supplementary Methods, Supplementary Fig. S2).

Subsequent analysis revealed a statistically significant up-regulation of HOTAIR among 15.89% of the CaCx cases (39.4-fold, p < 0.001), as compared to the HPV negative controls. Such up-regulation was 52.35-fold (p < 0.001) in HPV16 integrated cases and 28.2-fold (p = 0.007) in episomal cases. However, difference in HOTAIR expression between the episomal and integrated groups was not statistically significant. About 84.11% of the CaCx cases on the other hand, showed a consistent down-regulation of HOTAIR expression (6.77-fold, p < 0.001), as compared to the HPV negative controls. The episomal samples portrayed a down-regulation by 6.19-fold (p < 0.001) and integrated samples showed 7.78-fold down-regulation (p < 0.001), which did not differ significantly. Thus, HOTAIR deregulation in HPV16 positive CaCx cases was independent of the physical status of the viral genomes (Supplementary Fig. S3).

The LH and HH CaCx samples also harboured low and high levels of HPV16 E7 expression, respectively (Supplementary Fig. S3). The expression of E7 was significantly higher among CaCx cases (19.03-fold; p < 0.001) with HH as compared to the LH group, and this did not differ significantly between episomal (26.85-folds; p = 0.002) and integrated (10.6-folds; p = 0.004) CaCx cases. This observation identified that HOTAIR deregulation was probably correlated with HPV16 E7 expression, but independent of the viral integration status.

### HOTAIR expression in HPV16 positive CaCx cases shows a correlation with HPV16 E7 expression

**Figure 2:**
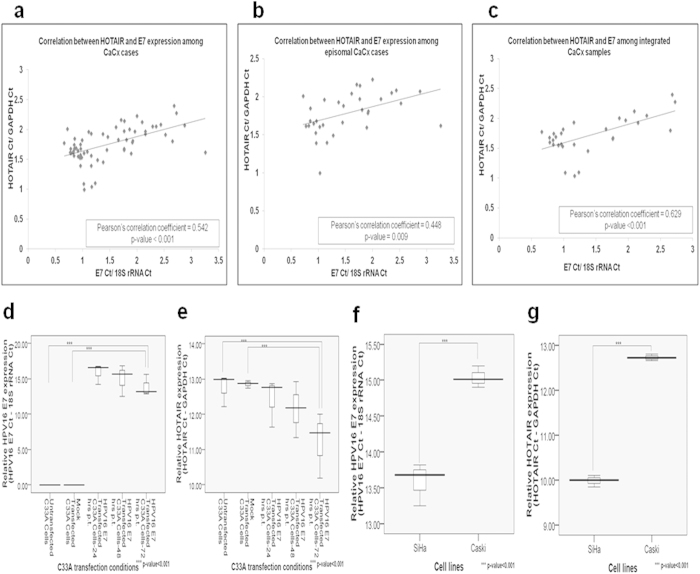
HOTAIR expression correlates positively with HPV16 E7 expression. (**a**–**c**) Correlation analysis between HOTAIR and HPV16 E7 expression among CaCx cases, episomal and integrated CaCx cases, respectively. (**d**,**e**) Box plots representing distribution of HPV16 E7 and HOTAIR expression levels respectively, in C33A cell line at various time points post-transfection (p. t.) of pcDNA3.1-HPV16 E7 vector. (**f**,**g**) Box plots representing distribution of expression levels of HPV16 E7 and HOTAIR among SiHa and Caski cell lines respectively.

We employed two approaches, to confirm the correlation between E7 and HOTAIR expression, based on both clinical samples and CaCx cell lines. HOTAIR expression was positively and significantly correlated with HPV16 E7 expression among the CaCx samples harbouring episomal or integrated HPV16 (Fig. 2a–c). Expression of HOTAIR and E7 correlated with viral load only among episomal CaCx cases (Supplementary Fig. S4). This was in concordance with an earlier report from our laboratory^36^ that identified similar correlation between E7 expression and viral load among CaCx cases with episomal HPV16, ensuring the expression of E7 from all episomal viral genomes. Taken together the observations point towards a possible dependence between HOTAIR and E7 expression.

In the second approach, we cloned HPV16 E7 into mammalian expression vector pcDNA3.1(+), and transfected this into the HPV negative CaCx cell line, C33 A. This resulted in a significant increase in HOTAIR expression concomitant with increase in HPV16 E7 expression (Fig. 2d–e). We also determined HOTAIR expression in HPV16 positive CaCx cell-lines, SiHa and Caski. HOTAIR expression was found to be higher among SiHa cells (Fig. 2f–g) that portray relatively higher E7 expression, as compared to Caski cells. Thus, both the clinical samples and cell line based observations confirmed that E7 could probably influence HOTAIR expression in CaCx cases.

### HOXD10, a target of HOTAIR, shows a statistically significant up-regulation in expression among CaCx cases

**Figure 3:**
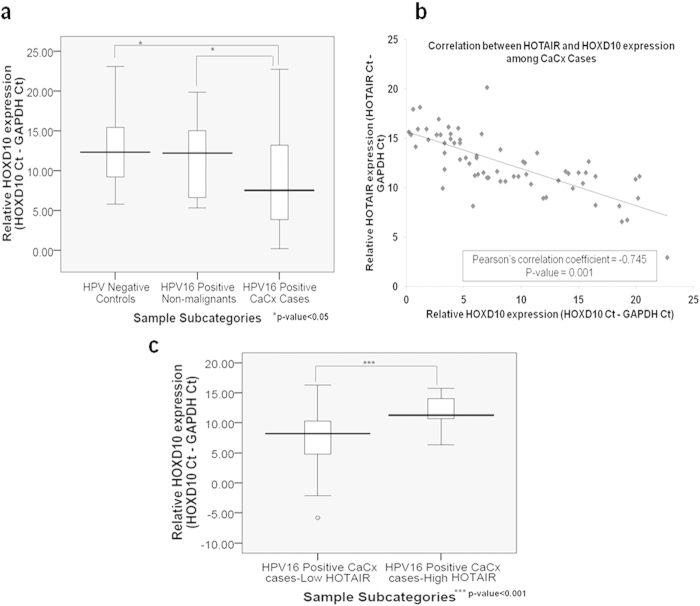
Up-regulation of HOXD10 expression correlates negatively with HOTAIR. (**a**) Box plots representing distribution of HOXD10 expression levels among the different categories of cervical samples. (**b**) Correlation analysis between HOTAIR and HOXD10 expression among the CaCx cases. (**c**) Box plots representing distribution of relative expression levels of HOXD10 among the low and high HOTAIR category of CaCx cases.

HOTAIR deregulation is likely to influence the expression of its downstream target genes through altered recruitment of PRC2-complex. Hence, we determined the expression of HOXD10, a known target of HOTAIR. It is a transcription factor, which has been reported to be deregulated in several cancer types^21^,^37^,^38^,^39^,^40^. We used a subset of samples selected for HOTAIR expression analysis comprising of CaCx cases (n = 70), HPV16 positive non-malignants (n = 11) and HPV negative controls (n = 19). HOXD10 expression was significantly higher among cases, irrespective of HPV16 physical status (integrated or episomal) and HOTAIR expression levels (LH or HH), with a fold-change of 27.5 (p = 0.012) as compared to HPV negative controls (Fig. 3a). The expression of HOXD10 also correlated negatively with HOTAIR expression among the CaCx cases (Fig. 3b), but not among the HPV negative controls. HOXD10 expression was significantly up-regulated among the LH CaCx cases (10.33-fold; p < 0.001) as compared to the HH CaCx cases (Fig. 3c). Such up-regulation was also recorded among the LH (48.5-fold; p = 0.0034) and HH (2.2-fold; p = 0.378) CaCx cases as compared to HPV negative controls, which was significant only among the LH cases. The findings potentially highlighted the inability of HOTAIR to *trans* regulate its target HOXD10, in E7 expressing CaCx cases.

### Gene expression profiling identifies distinct differences between LH and HH cases

**Figure 4:**
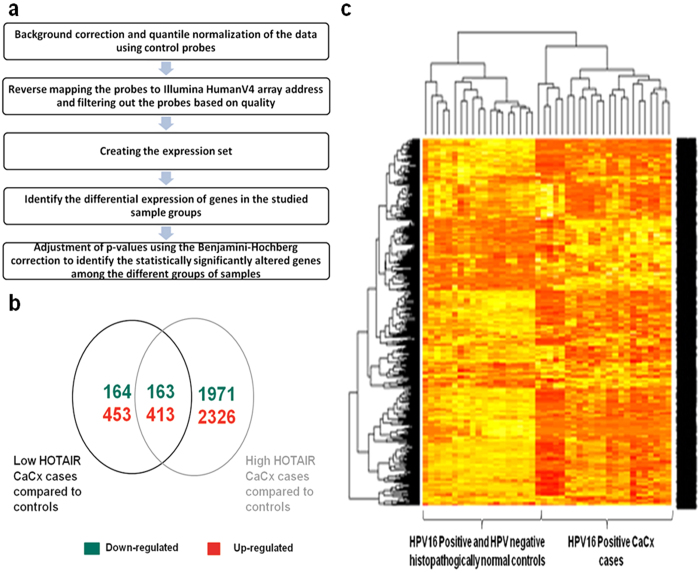
Microarray based global gene expression profiling. (**a**) Analysis pipeline utilised for identification of differentially expressed genes between sample groups. (**b**) Venn diagram depicting the number of genes differentially expressed among the low and high HOTAIR CaCx cases. (**c**) Hierarchical clustering distinctly separating the CaCx cases from the histopathologically normal controls (HPV negative and HPV16 positive) based on the differentially expressed genes.

The loss of HOXD10 silencing recorded above was suggestive of functional abrogation of HOTAIR in presence of E7, which could probably have an impact on global gene expression as well, among CaCx cases. Therefore, coupled with the identification of a subset of CaCx cases expressing high levels of HOTAIR compared to controls, it became essential to draw insights into the biological relevance of HOTAIR deregulation in CaCx cases. We therefore undertook global gene expression profiling using the Illumina HT-12_v4 gene expression array employing 11 HPV negative controls, 11 HPV16 positive non-malignants and 20 HPV16 positive cases comprising of 18 LH and 2 HH CaCx cases. The differentially expressed genes were subsequently identified and using the analysis pipeline illustrated in Fig. 4a, we identified the genes associated with disease development in the two categories of CaCx cases, as compared to the histopathologically normal controls.

Analysis revealed that 327 genes were down-regulated and 866 genes were up-regulated among the LH CaCx cases, while 2134 and 2739 genes (Fig. 4b) were down and up-regulated respectively, among the HH CaCx cases as compared to the histopathologically normal controls (HPV negative as well as HPV16 positive). Hierarchical cluster analysis could demarcate the CaCx cases from the histopathologically normal controls (Fig. 4c).

Further, we attempted to obtain a global view of the altered and distinct biological functions that could be associated with CaCx pathogenesis, based on levels of HOTAIR expression among the CaCx cases. We performed functional analysis on the sets of differentially expressed genes characteristic of LH and HH CaCx cases, respectively, employing Ingenuity Pathway Analysis (IPA) software. The LH CaCx cases were characterized by a number of cellular intrinsic processes such as Cell Death and Survival, Gene Expression, Cell Cycle, Cellular Assembly and Organization, Cellular Function and Maintenance, Tissue Development, DNA Replication, Recombination and Repair, etc. However, Cellular Growth and Proliferation (p = 1.28E-15) seemed to be the topmost biological process (Supplementary Table 1A–E) relevant for such cases, involving 236 genes, the maximum compared to the other biological processes involved. The HH CaCx cases were also characterized by cell intrinsic processes such as Cell Proliferation, Cell Cycle, Cellular Development, Cellular Growth, Cell Death and Survival, etc. However, such HH CaCx cases, were most prominently characterized by cell extrinsic processes such as Cellular Movement representing cell migration and invasion, and Cancer representing advanced malignant tumor, metastasis, breast or ovarian cancer, pelvic cancer, head and neck cancer etc.(Supplementary Table S1 A–E). The top significantly altered pathways are listed in Supplementary Table S2 A–E.

### PRC2-complex members, EZH2 and SUZ12, show E7-dependent increased expression in CaCx cases

HPV16 E7 harbours the ability to lower the global levels of H3K27 me3, which are known chromatin repressive marks, which facilitates global gene expression. Paradoxically, this has been recorded among E7 expressing cells, CaCx samples and cell lines, despite the high expression of EZH2, a PRC2-complex member^12^,^14^,^31^,^32^. HOTAIR-mediated PRC2-complex recruitment also creates H3K27 me3 marks through EZH2 enzymatic activity^21^,^25^,^30^. Therefore, we estimated the expression of both EZH2 and SUZ12 by qRT-PCR, to confirm the impact of E7 on PRC2 complex members in our clinical samples and CaCx cell lines transfected with E7.

**Figure 5:**
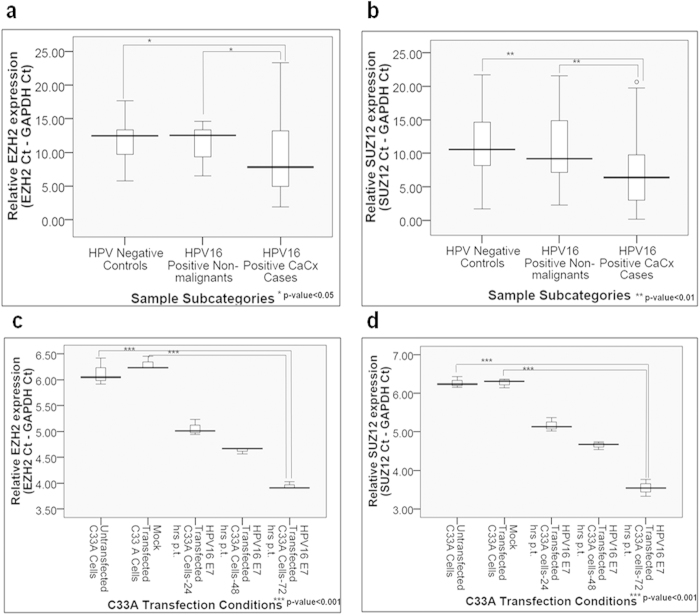
Increased expression of PRC2-complex members, EZH2 and SUZ12, among CaCx cases appears to be HPV16 E7-dependent. (**a**,**b**) Box plots representing distribution of EZH2 and SUZ12 expression levels respectively, among different categories of cervical samples. (**c**,**d**) Box plots representing distribution of EZH2 and SUZ12 expression levels in C33A cell line, at various time points post-transfection (p.t.) of pcDNA3.1-HPV16 E7 vector.

EZH2 and SUZ12 were both overexpressed among the CaCx cases by 23.4-fold (p = 0.034) and 30.6-fold (p = 0.0056) respectively, compared to HPV negative controls (Fig. 5a–b). We also identified a positive correlation of EZH2 and SUZ12 mRNA expression, with HPV16 E7 mRNA expression among the CaCx cases (Supplementary Fig. S5). In order to confirm the influence of E7 on the expression of PRC2 complex members, we employed E7 transfected C33 A cell line and evaluated the expression of EZH2 and SUZ12. E7 expression was found to be concomitant with increase in the expression of both the PRC2-complex members. While EZH2 was 4.54-folds up-regulated, SUZ12 showed an increase by 6.37-fold at 72 hours post-transfection, as compared to the untransfected cells. The changes were found to be statistically significant (p < 0.001) for both (Fig. 5c–d). These changes were also recorded at the protein level, as confirmed by immunoblot analysis (Supplementary Fig. S5). Thus, we confirmed the potential role of E7 in modulating expression of the PRC2-complex members in CaCx cases. Our finding appeared to be in line with previous reports^30^,^31^,^32^.

### HPV16 E7 possesses the ability to interact with HOTAIR

**Figure 6:**
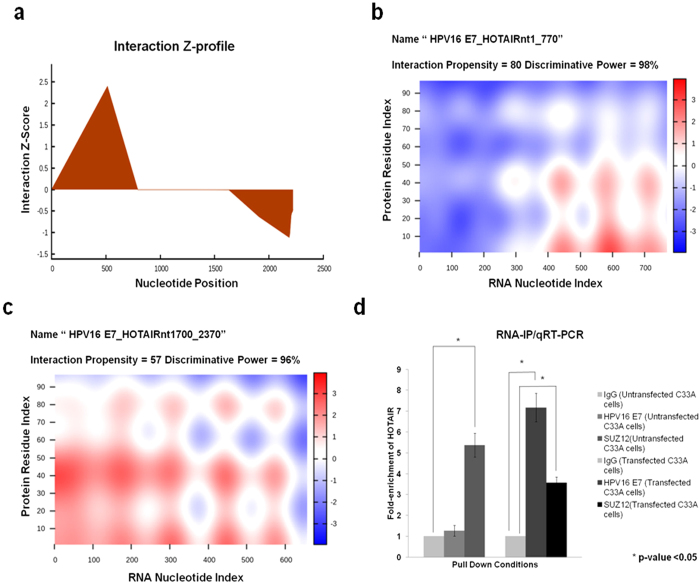
HOTAIR interacts with HPV16 oncoprotein E7. (**a**) catRAPID fragments based prediction of interaction between HOTAIR and HPV16 E7 and maps HOTAIR interaction domains to nts 1–770 (5′-end) and nts 1700–2370 (3′-end). (**b**,**c**) catRAPID graphic output confirming the interaction of HPV16 E7 to the 5′ and 3′ ends of HOTAIR transcript as predicted by catRAPID fragments. (**d**) qRT-PCR results shows HOTAIR is significantly enriched with the SUZ12 antibody compared to IgG (control antibody) in untransfected C33A cells while a significant enrichment of HOTAIR was found both for HPV16 E7 and SUZ12 antibody in HPV16 E7 expressing C33A cells although lower for SUZ12.

We further tested the hypothesis that the reported E7 mediated reduction in global H3K27 me3 marks^31^,^32^, in presence of high levels of PRC2 complex members, could be the result of interference with HOTAIR function. To confirm this, we first used an *in silico* approach to identify the possibility of any physical interaction between E7 and HOTAIR, based on two RNA-protein interaction prediction tools: RPIseq (RNA-protein interaction prediction software) and catRAPID (a software tool trained using known lncRNA-protein interactions, and used specifically to predict lncRNA-protein interactions). Both the tools identified a strong propensity of interaction between HOTAIR and HPV16 E7. RPISeq predicted a strong interaction between the two, giving an RF classifier value of 0.8 and SVM classifier value of 0.77. To identify, which region of HOTAIR possessed the ability of interaction with HPV16 E7, we used catRAPID fragments algorithm. This tool identified both the 5′ and the 3′ ends of HOTAIR transcript to possess interaction propensities with HPV16 E7 (Fig. 6a). catRAPID graphic also confirmed the results of catRAPID fragments and identified nucleotides 1–770 (5′-region) and 1700–2370 (3′-region) of HOTAIR to show interaction propensities of 80 and 57 respectively, and very high discriminative powers of 98% and 96% respectively (Fig. 6b–c). The predicted interactions highlighted regions of HOTAIR, which are known to interact with PRC2-LSD1 chromatin remodelling complex.

To confirm if HPV16 E7 interacted physically with HOTAIR, we used RNA-Immunoprecipitation (RIP), employing HPV16 E7 antibody followed by qRT-PCR. This showed enrichment of HOTAIR (7.17-fold), as compared to RIP with IgG antibody (negative control), in HPV16 E7 expressing C33 A cells. SUZ12 antibody based RIP also showed a significant enrichment of HOTAIR in untransfected C33 A cells (5.37-fold), as well as HPV16 E7 expressing C33 A cells (3.56-fold), which was lower in the latter (Fig. 6d, Supplementary Fig. S6). This observation provided strong evidence that HPV16 E7 physically interacted with HOTAIR. Hence, we predicted that such interaction probably excluded PRC2-complex from the HOTAIR binding-site, thereby affecting PRC2-complex recruitment and subsequent loss of gene silencing through reduction of H3K 27me3 levels.

## Discussion

In this study, our focus was to decipher the involvement of lncRNA HOTAIR, a regulator of gene expression through PRC2 recruitment, in CaCx pathogenesis. In view of the causal relevance of the viral oncoprotein E7 in CaCx pathogenesis, we further explored the interplay between E7 and HOTAIR in order to explore if HOTAIR could be targeted by E7 to achieve up-regulation of cancer related pathway genes. We determined the expression levels of HOTAIR and E7 in various categories of cervical tissue samples and cell lines. Employing *in silico* analysis and RNA-Immunoprecipitation assay, we interrogated the likelihood of physical interaction between the two.

Our study revealed a significant linear trend of progressive HOTAIR down-regulation through the discrete stages of CaCx development and its expression was E7-dependent. During early infection, E7 has been identified to create a milieu conducive for HPV16 DNA synthesis, by perturbing the keratinocyte differentiation program and inducing host DNA replication^41^. This could possibly be achieved through lowering of HOTAIR expression by low levels of E7, thereby prompting low levels of up-regulation of HOTAIR target genes, including, HOXD10. HOXD10 has been reported to be negatively correlated with rate of histologic differentiation in endometrial^42^ and breast cancers^43^. Hence, HOTAIR deregulation, under the impact of low levels of E7, could be reflective of gene expression deregulation as well, in early infection. This could perhaps facilitate an abortive life cycle leading to HPV16 persistence. Therefore, HOTAIR could probably serve as an early marker for singling out HPV16 positive women at risk of developing CaCx in the long run, for which further studies are warranted.

Although the observed down-regulation among the HPV16 positive CaCx cases was contradictory to the prevailing concept of HOTAIR up-regulation in most cancers^21^,^26^,^27^,^28^,^29^, including a couple of reports on cervical cancers^33^,^34^, almost all such reports reveal the existence of two subgroups. In most of these reports, 70–80% of the cancer samples harbour low HOTAIR expression, while 20–30% of the cancer samples show high HOTAIR expression. However, the reports on CaCx^33^,^34^ differed with respect to the proportion of samples with low and high HOTAIR expression. While one study portrayed equal proportions^33^, the other^34^ identified proportions similar to that recorded by us where 84% and 16% of the CaCx samples portrayed LH or HH expression, respectively. But none of these studies considered the impact of HPV on HOTAIR deregulation. Our study design also differed from one of the studies^33^ distinctly, as we avoided the use of cancer adjacent normal tissues as controls. In such controls, there is a high probability of the presence of HPV infection at relatively high viral load, if not to the levels observed in cancer cells. Instead, we used normal samples that were HPV negative and included the HPV16 positive histopathologically normal tissues, as an intermediate group. This seemed to be biologically relevant in the context of viral load and E7 expression, which are established to be higher in CaCx cases, compared to HPV16 positive non-malignant samples^44^.

The correlation between E7 and HOTAIR expression among CaCx cases and cell lines was indicative of the role of E7 in regulating HOTAIR expression. However, the probable mechanism of this regulation remains unclear and demands further study. A recent study, however, recorded down-regulation of HOTAIR expression in prostate cancer cells mediated by miR-34a^45^ and this miRNA has been reported to be differentially expressed in CaCx cases as well. The interplay between viral oncoproteins E6, E7 and the host encoded p53 is known to regulate miR-34a expression^46^. Thus, HOTAIR down-regulation among the CaCx cases could be brought about by E7-mediated gene regulatory mechanisms through miR-34a, which needs experimental validation.

HOTAIR down-regulation was concomitant with HOXD10 up-regulation among the CaCx cases, in our study. HOXD10 is a tumor suppressor and is known to be down-regulated in a variety of cancer types, exceptions being early stage HNSCC and oral squamous cell carcinoma, where HOXD10 is up-regulated^39^,^40^. We are the first to report HOXD10 up-regulation in HPV16 positive CaCx cases, in the context of HOTAIR down-regulation. This observation appears to be similar to that of Hakami *et al.*, for HNSCCs^39^. The latter study showed that HOXD10 overexpression resulted in decreased cellular invasion and increased the proliferation and adhesion abilities of the cells, while knockdown of HOXD10 revealed opposite results. Such studies, including ours, reflected that increased HOXD10 in HPV16 positive CaCx cases provided a proliferative advantage to the cells.

Global gene expression profiling also identified an up-regulation of a large number of genes and revealed enrichment of a number of biological processes associated with cancer related mechanisms such as, Cell Growth and Proliferation, Cell Death and Survival, etc. among the LH and HH subtypes of CaCx cases. However, mechanistically the HH cases seemed to be distinct from the LH cases. The HH cases portrayed enrichment of processes like Cellular Movement involving migration and movement of cells, as well as, Cancer Related Processes associated with advanced malignant tumours and metastasis. A distinct set of genes and pathways were also found to be differentially up-regulated among both categories of CaCx cases, LH and HH. These included Cell Cycle: G2/M DNA Damage Checkpoint Regulation, Cell Cycle Control of Chromosomal Replication, p53 Signaling, ATM Signaling, and Role of BRCA1 in DNA Damage Response etc. However, the HH CaCx cases appeared to be distinct from the LH cases in terms of genes and pathways associated with metastasis such as, Agranulocyte Adhesion and Diapedesis, Granulocyte Adhesion and Diapedesis, Glioma Invasiveness Signaling etc., which were not identified among LH cases. Thereby, we confirmed the metastatic potential of high levels of HOTAIR in HPV16 positive CaCx cases, a finding similar to reports on other cancers^21^,^26^,^27^,^28^,^29^. Based on such analysis, we further speculated that the HH and LH cases were likely to be prognostically distinct, warranting the need for clinical correlation in subsequent studies. However, we failed to record any association between HOTAIR levels (LH or HH) and tumour stage or histopathological grades of the CaCx cases. This could probably be attributed to the homogeneous nature of our CaCx samples, which were all SCCs, majority being moderately differentiating and non-keratinizing types, with tumour stage III and above. Our findings have therefore enriched the body of literature pertaining to the identification of molecular subtypes within various cancers, further justifying HOTAIR expression deregulation in HPV16 positive CaCx cases as a potential diagnostic marker.

Interestingly, we noted that irrespective of the status of HOTAIR expression, there was an up-regulation of a large number of genes in CaCx cases, which is expected if there is E7-mediated reduction of H3K27 me3 marks, as reported previously^31^,^32^. Such reduction in the levels of H3K 27me3 in HPV16 positive cervical lesions and HPV16 E7 expressing cells has been attributed to induced KDM6A and KDM6B expression by Munger *et al.*^12^,^31^. But among the CaCx cases, this reduction occurs in presence of an E7-dependent increased expression of PRC2-complex members. This was suggestive of a probable functional impairment of HOTAIR in PRC2-complex recruitment and subsequent loss of gene silencing marks, causing up-regulation of a variety of cancer related pathways. Our speculation was further strengthened based on (i) *in silico* analysis revealing binding sites for E7 within the 5′ and 3′ domains of HOTAIR, which needs further validation and (ii) RIP/qRT-PCR confirmed physical interaction between HOTAIR and E7.

**Figure 7:**
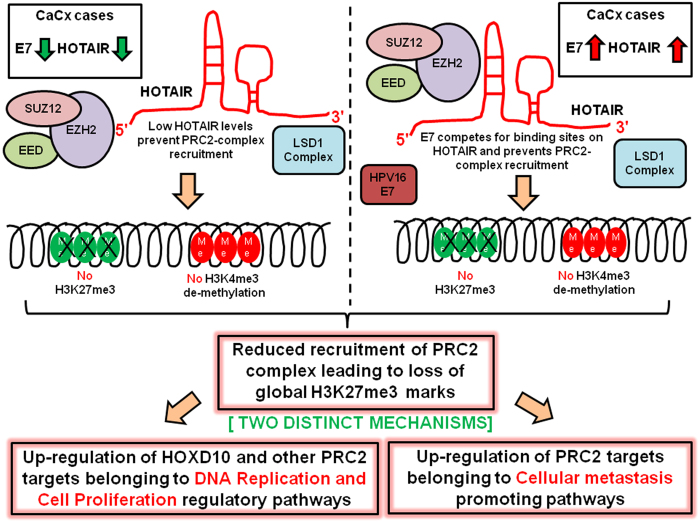
Schematic illustration of E7-mediated regulation of global gene expression through HOTAIR by altering PRC2 activity.

Thus, two distinct mechanisms could be responsible for the global gene expression up-regulation among the CaCx cases as indicated in Fig. 7. Among the LH cases, the reduced availability of HOTAIR transcripts for the recruitment of the PRC2-complex could be leading to reduced global H3K27 me3 marks and subsequent up-regulation of the target genes. Among the HH cases, the abundant oncoprotein E7 could be competing with highly expressed PRC2-complex members for binding sites on the HOTAIR transcript. This could prevent the recruitment of PRC2-complex, thereby facilitating up-regulation of the target genes. Our study is the first of its kind to depict that one of the causal mechanisms of cervical carcinogenesis mediated by HPV16 E7, was through modulation of HOTAIR expression and function. HOTAIR therefore appeared to be one of the many host molecules targeted by HPV16 E7, in cervical carcinogenesis.

In conclusion, we provided evidence of interplay between HPV16 E7 oncoprotein and lncRNA HOTAIR in regulating the PRC2-mediated global gene expression machinery, concomitant with the cellular processes involved in cell proliferation and metastasis. HPV16 E7, therefore seems to be a master regulatory molecule, facilitating CaCx development through this novel HOTAIR mediated regulation. This is a first glimpse towards understanding the molecular events critical to global transcriptional reprogramming, involving the interaction between a viral oncoprotein and lncRNA. Further studies employing chromatin Immunoprecipitation and deep sequencing (ChIP-Seq) for determining chromatin repressive marks (H3K27 me3) and activation marks (H3K4 me3) that are regulated by PRC2 and LSD-1 complexes respectively, are underway. Such data, together with our gene expression profiling data, could help in the delineation of gene sets that have altered gene silencing and activation marks at low and high HOTAIR levels. This could provide insights on disease pathogenesis by identification of markers related to disease aggressiveness and prognosis. Overall, the study holds potential of providing guidelines for the development of prevention and therapeutic strategies for combating such cancers.

## Methods

### Sample Collection, DNA Isolation and HPV testing

The CaCx samples used for this study were derived from married subjects aged 28–80 years (median: 52 years) attending a cancer referral hospital (Saroj Gupta Cancer Centre and Research Institute, South 24 Parganas, West Bengal , India). All malignant samples were histopathologically confirmed as invasive squamous cell carcinomas (majority were diagnosed as non-keratinizing and moderately differentiated squamous cell carcinoma pathologically) as per WHO classification and clinically diagnosed as stage III and above as per FIGO classification. The healthy control tissues were derived from married women aged 25–75 years (median: 48 years) from Calcutta Medical College and Hospital, Kolkata, West Bengal, India. There was no significant difference between the median age of the CaCx cases and the non-malignants. All control samples (collected from individuals without any prior history of cervical dysplasia/malignancy) were histopathologically confirmed as non-malignant and after testing for HPV were classified as HPV negative or positive. Of the HPV positive samples we selected only those with HPV16. Samples showing presence of HPV18 along with HPV16 were excluded from the study.

All samples, cases and non-malignant samples were collected from the subjects with informed consent approved by the ethical committee of National Institute of Biomedical Genomics for human experimentation. Details regarding DNA isolation, HPV screening and determination of HPV16, and physical status of HPV16 genomes have been described in details earlier from our laboratory^35^,^47^,^48^,^49^.

### Plasmids and clones

Mammalian expression vector pcDNA3.1(+) was purchased from Invitrogen (Cat # V790-20) and used for cloning of HPV16 E7 ORF. HPV16 E7 region was amplified with primers (Forward Primer: GGAGGTACCGTCATGCATGGAGATACACCTACA, Reverse Primer: GAGGCGGCCGCTTTATGGTTTCTGAGAACAGAT) harbouring restriction sites for subsequent cloning of the amplified fragment into pcDNA3.1(+). The amplified fragments and the vector DNA were digested using restriction enzymes NotI (NEB, Cat# R0189S) and KpnI (NEB, Cat# R0142S) and the digested products were ligated using T4 DNA Ligase (Roche, Cat# 10716359001) by incubating overnight at 16 °C. The plasmid pcDNA3.1-HPV16 E7 was checked for insertion of the E7 fragment into the mammalian expression vector by Sanger sequencing.

### Cell Culture and transfection

HPV negative cell line C33 A (Cervical carcinoma cell line derived from a 65-year old Caucasian female with cells being devoid of HPV DNA or RNA, http://www.atcc.org/products/all/HTB-31.aspx) was obtained from the laboratory of Professor Sudhir Krishna (Cellular Organisation and Signalling, National Centre for Biological Sciences, Bangalore, India) and was cultured in DMEM (Gibco, Cat# 11995-065) supplemented with 10% FBS (Gibco, Cat# 16000-044), 50 Units/ml of penicillin and 50 μg/ml of streptomycin (Gibco, Cat# 15070-063) at 37 °C and 5% CO_2_. C33 A cells were transfected using Lipofectamine 2000 reagent (Invitrogen, Cat# 11668-027) as per the manufacturer’s protocol, using 1 μg of plasmid pcDNA3.1-HPV16 E7. The cells were harvested at three time points: 24, 48 and 72 hrs post-transfection of cells with HPV16 E7. Empty vector pcDNA3.1(+) was used for mock. The cells were washed with 1X PBS, pH 7.4 (Gibco, Cat# 14190-144), trypsinized and collected by centrifugation at 300 g for 10 minutes. The transfected cells were used further for RNA and protein isolation. The transfection experiments were carried out in three sets, each in triplicates. The cells were monitored for mycoplasma contamination, employing standard protocols.

### RNA Isolation and reverse transcription

RNA isolation methods and generation of cDNA using random hexamers and a combination of Oligo-dTP3 and random hexamers, are described in Supplementary Methods.

### Identification of the physical status of HPV16 and estimating the viral load

Confirmation of the integration or episomal status of HPV16 was carried out using APOT-coupled Taqman assay, as described previously^36^. Viral load was also estimated by Taqman assay, as described in earlier reports^44^.

### Microarray based transcriptome profiling

RNA isolated from tissue samples was checked for integrity, using Agilent 2100 Bioanalyzer and Agilent RNA 6000 Nano reagents (Cat# 5067-1512). Only samples with RNA Integrity Number (RIN) of 6.0 and above were selected for the transcriptome analysis. Gene expression profiling was hence carried out on 20 CaCx case samples (18 low HOTAIR category and 2 high HOTAIR category), 11 non-malignant HPV 16 positive samples and 11 HPV Negative Controls using the Illumina Human HT-12 v4 Expression Bead Chip Kit (Cat# BD-103-0204) and the scanning of the arrays was done, using the Illumina iScan Microarray scanner. The data has been submitted in the GEO database, NCBI (Accession No: GSE67522).

Gene expression profiles for all the categories of cervical samples were generated using one-color hybridization to whole human genome arrays, using Illumina’s 47,231 probes. The probe quality of the array was assessed before and after normalization and the background correction was done. Appropriate batch effects correction was done. To improve data quality, a filtering of the probes was applied. The probes containing repetitive sequences, binding to multiple sites of human transcriptome, were removed for further analysis. Downstream analysis was done to identify the differentially expressed genes, based on t-test among both the categories (low and high HOTAIR) of CaCx cases compared to histopathologically normal controls (HPV negative controls and HPV16 positive non-malignant samples). We failed to record any differentially expressed genes, between HPV negative controls and HPV16 positive non-malignants. The p-values were determined and multiple testing corrections (Benjamini Hochberg method) done to remove the false discovery rate. The differentially expressed genes were selected on the basis of adjusted p < 0.05 and fold change of gene expression, compared to histopathologically normal controls (for up-regulation, fold change ≥1.5 and for down-regulation, fold change ≤−1.5).

To obtain a global view of the altered and distinct biological functions, functional analysis employing Ingenuity Pathway Analysis software (IPA) was done on the set of differentially expressed genes mentioned above. IPA is known to provide more robust results, rather than studying individual genes. The top biological processes were identified among the low and high HOTAIR expressing CaCx samples, to identify any mechanistic differences between the two categories.

### Expression analysis using qRT-PCR

HOTAIR, E7, EZH2, SUZ12 and HOXD10 gene expressions were quantified in, CaCx samples with episomal (pure or concomitant) or integrated HPV16 (pure integrated) HPV16, non-malignant HPV 16 positive samples and HPV negative controls. Expression analysis was done by qRT-PCR, on ABI 7900HT by relative quantification, using the comparative C_T_ method. Glyceraldehyde-3-phosphate Dehydrogenase (GAPDH) was chosen as an internal control for normalization. For all the samples, the median C_T_ –values for the target genes and GAPDH was taken and the expression of target genes was normalized with that of GAPDH. 18S rRNA (Life Technologies TaqMan® Gene Expression Assay, Cat# 4352930E) served as the normalization control, for E7 expression estimation. The dissociation curves and the amplification plots are provided in Supplementary Fig. S7. The primers used for the relative quantification are provided in Table 1.

### *In silico* prediction of lncRNA-protein interaction

*In silico* prediction was done to identify possible interaction between HOTAIR and HPV 16 E7 oncoprotein, using two RNA-protein interaction predictor tools, RPI-Seq (http://pridb.gdcb.iastate.edu/RPISeq/) and catRAPID (http://s.tartaglialab.com/page/catrapid_group).

### RNA-Immunoprecipitation (RIP) and qRT-PCR

C33 A (10^7^ Untransfected and HPV 16 E7 Transfected) cells were harvested by trypsinization and re-suspended in 2 ml PBS, 2 ml nuclear isolation buffer (1.28 M sucrose; 40 mM Tris-HCl pH 7.5; 20 mM MgCl2; 4% Triton X-100), and 6 ml water on ice, for 20 min (with frequent mixing). Nuclei were pelleted by centrifugation at 2,500 g for 15 min. Nuclear pellet was re-suspended in 1 ml RIP buffer (150 mM KCl, 25 mM Tris pH 7.4, 5 mM EDTA, 0.5 mM DTT, 0.5% NP-40, Protease Inhibitor Cocktail, 100 U/ml RNaseOUT Ribonuclease Inhibitor; Life Technologies). Re-suspended nuclei were split into two fractions of 500 μl each (for Mock and IP) and were mechanically sheared using a Dounce homogenizer with 15–20 strokes. Nuclear membrane and debris were pelleted by centrifugation at 13,000 rpm, for 10 min. Antibody (5μg) to HPV16 E7 or SUZ12 was added to supernatant for pull down along with mock controls and incubated for 2 hr at 4 °C, with gentle rotation. Then 40 μl of Protein A Agarose (Pierce, Cat# 20333) beads were added and incubated for 1 hr at 4 °C, with gentle rotation. Beads were pelleted at 2,500 rpm for 30 s, the supernatant was removed, and beads were re-suspended in 500 μl RIP buffer. This was repeated for a total of three RIP washes, followed by one wash in PBS. Beads were re-suspended in 1 ml of Trizol. The immunoprecipitated proteins were confirmed by western blot and the co-precipitated RNAs were isolated, followed by HOTAIR quantification by SYBR green qRT-PCR.

### Statistical Analysis

Kolmogorov-Smirnov test was done to check for the normality of the qRT-PCR data. The changes in expression levels were tested for statistical significance using non-parametric test (Mann-Whitney U Test) for groups not showing normal distribution. For experimental sets showing normal distribution, t-test was used to compare the means of the different sets. Multiple sets of experiments using cell lines, were compared, using ANOVA for statistically significant differences in expression levels between the experimental categories. All statistical analyses was done using SPSS 16.0 and R package.

## Additional Information

**How to cite this article**: Sharma, S. *et al.* Bridging Links between Long Noncoding RNA HOTAIR and HPV Oncoprotein E7 in Cervical Cancer Pathogenesis. *Sci. Rep.* **5**, 11724; doi: 10.1038/srep11724 (2015).

## Supplementary Material

Supplementary Information

## Figures and Tables

**Table 1:** Primer sequences for gene expression analysis using qRT-PCR.

**Target Name**	**Primer Sequence (5′- 3′)**	**Product Length (bps)**
**HOTAIR**	F: GGT CCT GCT CCG CTT CGC AG	116
	R: ACG CCC CTC CTT CCT CTC GC	
**HOXD10**	F: GCA GGA GAA GGA AAG CAA AGA GGA A	159
	R: CGC TCG CGG GTG AGG TAC AT	
**EZH2**	F: CCC TGA CCT CTG TCT TAC TTG TGG A	119
	R: CGT CAG ATG GTG CCA GCA ATA	
**SUZ12**	F: AAA CGA AAT CGT GAG GAT GG	115
	R: CCA TTT CCT GCA TGG CTA CT	
**GAPDH**	F:CAGCCTCAAGATCATCAGCA	106
	R:TGTGGTCATGAGTCCTTCCA	
**HPV16 E7**	F: AAG TGT GAC TCT ACG CTT CGG TT	78
	R: GCC CAT TAA CAG GTC TTC CAA A Probe: (6-FAM) TGC GTA CAA AGC ACA CAC GTA GAC ATT CGT A (MGB)	
