# Supplementary material for: Bridging Links between Long Noncoding RNA HOTAIR and HPV Oncoprotein E7 in Cervical Cancer Pathogenesis
Source: Sci Rep. 2015 Jul 8;5:11724. doi: 10.1038/srep11724 (PMC4495428; doi:10.1038/srep11724)
Supplement: Supplementary Information [file srep11724-s1.pdf]

Bridging Links between Long Noncoding RNA HOTAIR and HPV Oncoprotein E7 in Cervical Cancer Pathogenesis

Sweta Sharma[1], Paramita Mandal[1], Tamal Sadhukhan[1], Rahul Roy Chowdhury[2], Nidhu Ranjan Mondal[2], Biman Chakravarty[2], Tanmay Chatterjee[2], Sudipta Roy[3] and Sharmila Sengupta[1*]

[1]National Institute of Biomedical Genomics, Netaji Subhas Sanatorium, 2nd Floor, P.O.: N.S.S, Kalyani 741251, West Bengal, India

[2]Department of Gynecology, Saroj Gupta Cancer Centre and Research Institute, Kolkata, India.

[3]Sri Aurobindo Seva Kendra, 1H, Gariahat Road (S) Jodhpur Park, Kolkata - 700068, West Bengal, India

[*]Corresponding author

Supplementary methods

RNA Isolation and reverse transcription

Total RNA was isolated using ~20 mg tissue sections stored in RNA Later using the Qiagen RNeasy Kit (Cat # 74104) following manufacturer's protocol. RNA was quantified using the Qubit® RNA BR Assay Kit (Life Technologies, Cat # Q10211). 400 ng of RNA was used for reverse transcription using High Capacity cDNA Reverse transcription Kit with RNase Inhibitor (Cat # 43749666) in a 20 µl reaction using two different sets of primers. One set of cDNA was generated using Random Hexamers supplied with the kit and the other set was prepared using a mix of Random Hexamers and dT(17)-P3 primer. All the rest of the reagents for the reverse transcription reaction were added as per manufacturer's instructions. The reaction mix was incubated at 25°C for 10', followed by incubation at 37°C for 2 hours and heat inactivation of enzyme at 85°C.

ROC Curve Analysis

ROC Curve was created using the SPSS version 16.0 with the relative expression values (HOTAIR Ct – GAPDH Ct) of HOTAIR among case samples as the input for sample categorisation. The area under the curve (AUC) observed for the plot was 0.638 with a standard error of 0.055 (p-value=0.012). Then the point showing the best possible combination of sensitivity (0.879) and 1-specificity (0.595) was selected as the cut-off point to group the CaCx samples into high and low HOTAIR expressing sub-categories. The ROC curve is provided in the Supplementary Fig. S4. Although ROC analysis is a good tool for

bilateral distribution of a set of values, there are certain limitations of this analysis which include the requirement of large sample size to allow for the effects to be real and significant.

Immuno Blot analysis

Homogenization of the tissue samples (~ 10 mg) was done in 100 µl ice cold protein lysis buffer (30 mM Tris HCl; pH=7.5,1 mM $MgCl_2$, 1 mM EGTA,0.67% β-mercaptoethanol,0.5% CHAPs,10% Glycerol and 0.5% Triton X100). After overnight incubation at 4°C in shaking condition, and subsequent centrifugation at 12,000 rpm at 4°C for 20 minutes, the supernatant was collected. The protein concentration was determined using Bradford assay. 30 µg of protein samples were run on 12.5% SDS PAGE in duplicate and then the proteins were transferred onto 0.45 µm PVDF membrane. After blocking with 5% skimmed milk, the membrane was treated with 1:200 dilution of EZH2 primary antibody (Santa Cruz Biotechnology, sc-25383), 1: 200 dilution of SUZ12 primary antibody (Santa Cruz Biotechnology, sc-67105), 1:200 dilution of E7 primary antibody (Santa Cruz Biotechnology, sc-6981) overnight at 4°C. After washing, the membrane was again treated with respective secondary antibodies (1:5000 dilution, goat anti-mouse IgG-HRP, Santa Cruz Biotechnology, sc-2005 or goat anti-rabbit IgG-HRP, Santa Cruz Biotechnology, sc-2030) at 37°C for 2 hours. Protein expression was detected by chemiluminiscence based assay after washing the membrane. Expression of GAPDH was taken as loading control. Mouse monoclonal GAPDH primary antibody (1:5000 dilution, Abcam, ab9485) and anti-mouse secondary antibody (1:5000 dilution, goat anti-mouse IgG-HRP, Santa Cruz Biotechnology, sc-2005) were used for GAPDH protein expression study. Densitometric analysis of the western blot results were performed using ImageLab Software (BioRad).

Supplementary Figures

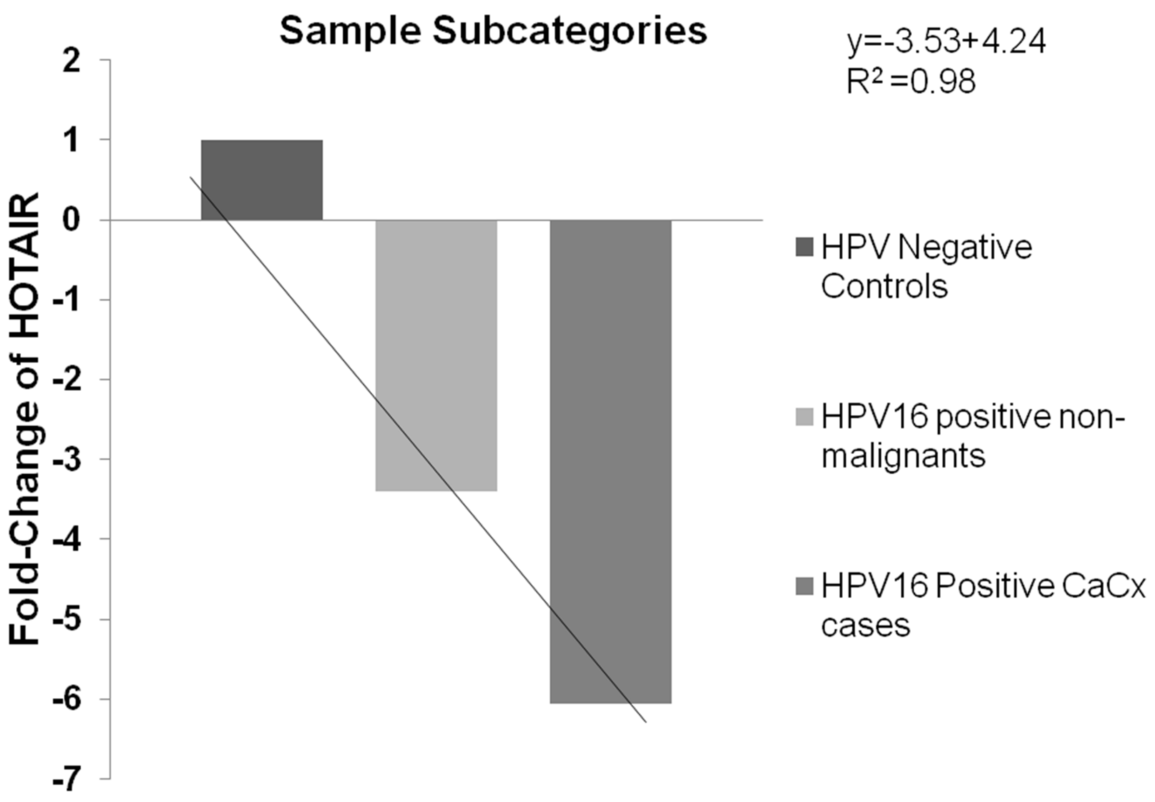

Supplementary Fig. S1| Linear regression analysis identifies a significant decreasing trend of HOTAIR expression (p-value = 0.034) from HPV negative controls to HPV16 positive non-malignants followed by HPV16 positive CaCx cases.

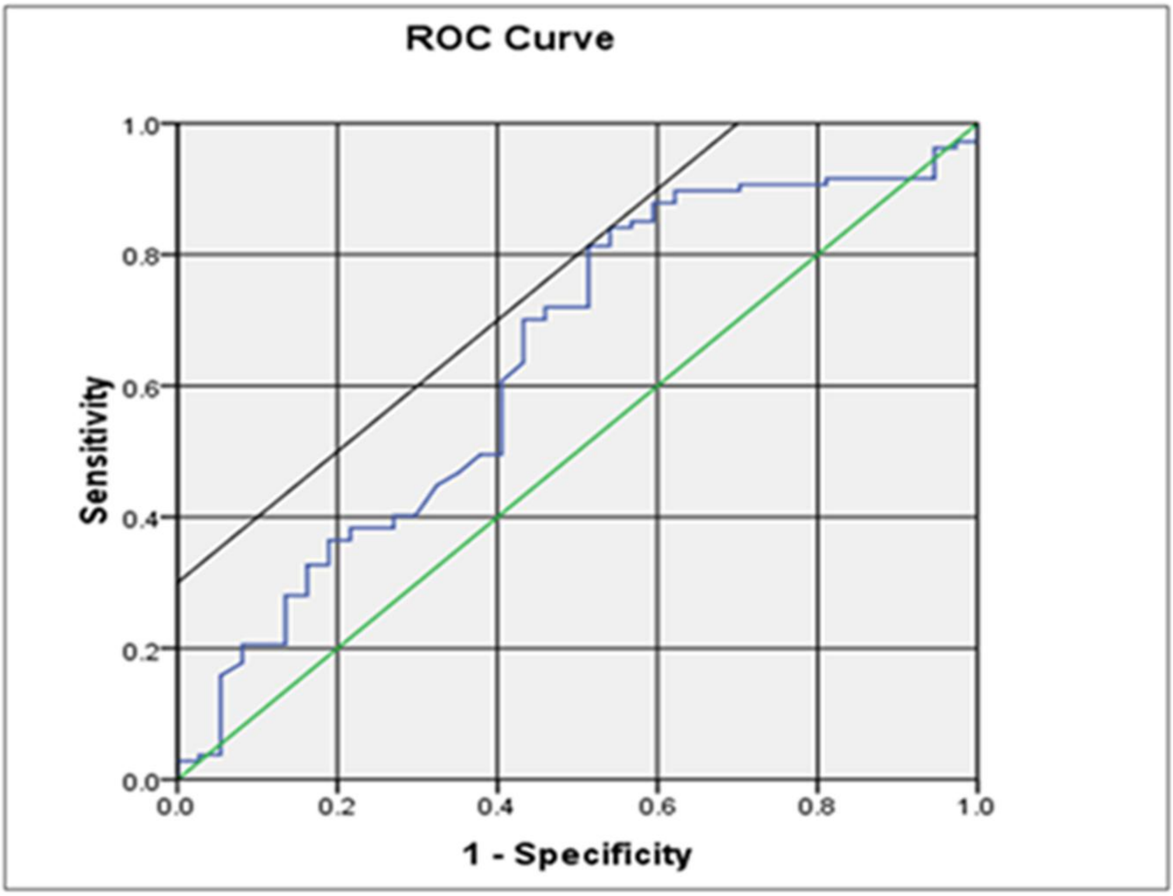

Supplementary Fig. S2| ROC analysis to identify subcategories among the CaCx cases based on their HOTAIR expression levels. The green diagonal line represents a specificity and sensitivity of 0.5 each. The black line identifies the point of intersection that can be utilised to classify samples at maximum specificity and sensitivity possible.

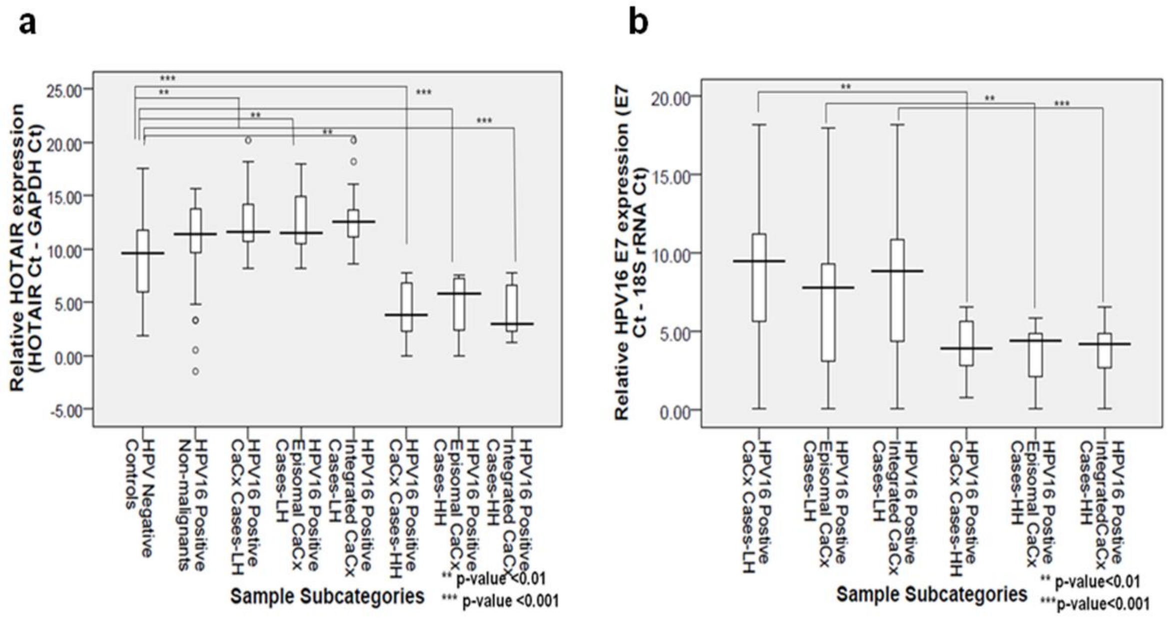

Supplementary Fig. S3| (a). & (b). Box plots representing distribution of HOTAIR and HPV16 E7 expression levels among ROC based subcategories of CaCx cases (LH=Low HOTAIR, HH=High HOTAIR).

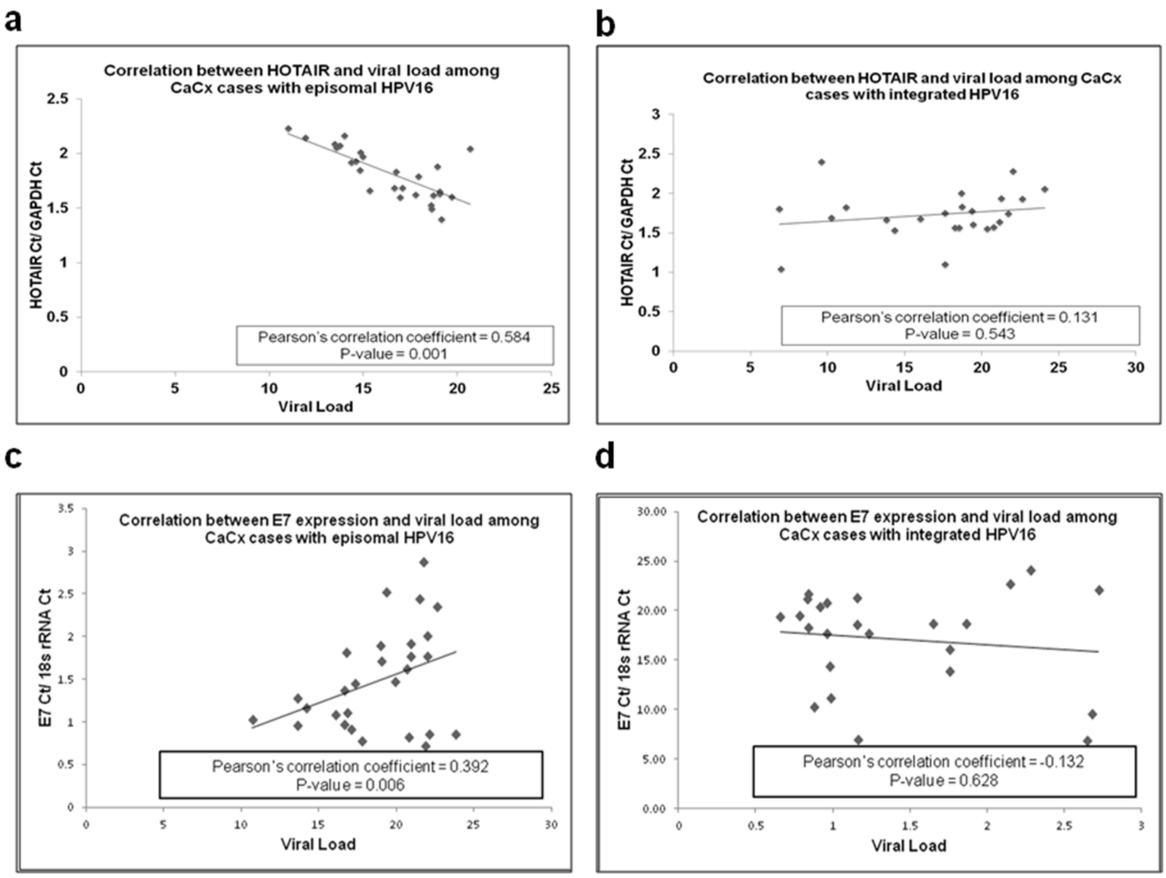

Supplementary Fig. S4: (a). & (b). Correlation analysis between HOTAIR expression and viral load among episomal and integrated CaCx cases, respectively. (c). & (d). Correlation analysis between HPV16 E7 expression and viral load among episomal and integrated CaCx cases, respectively.

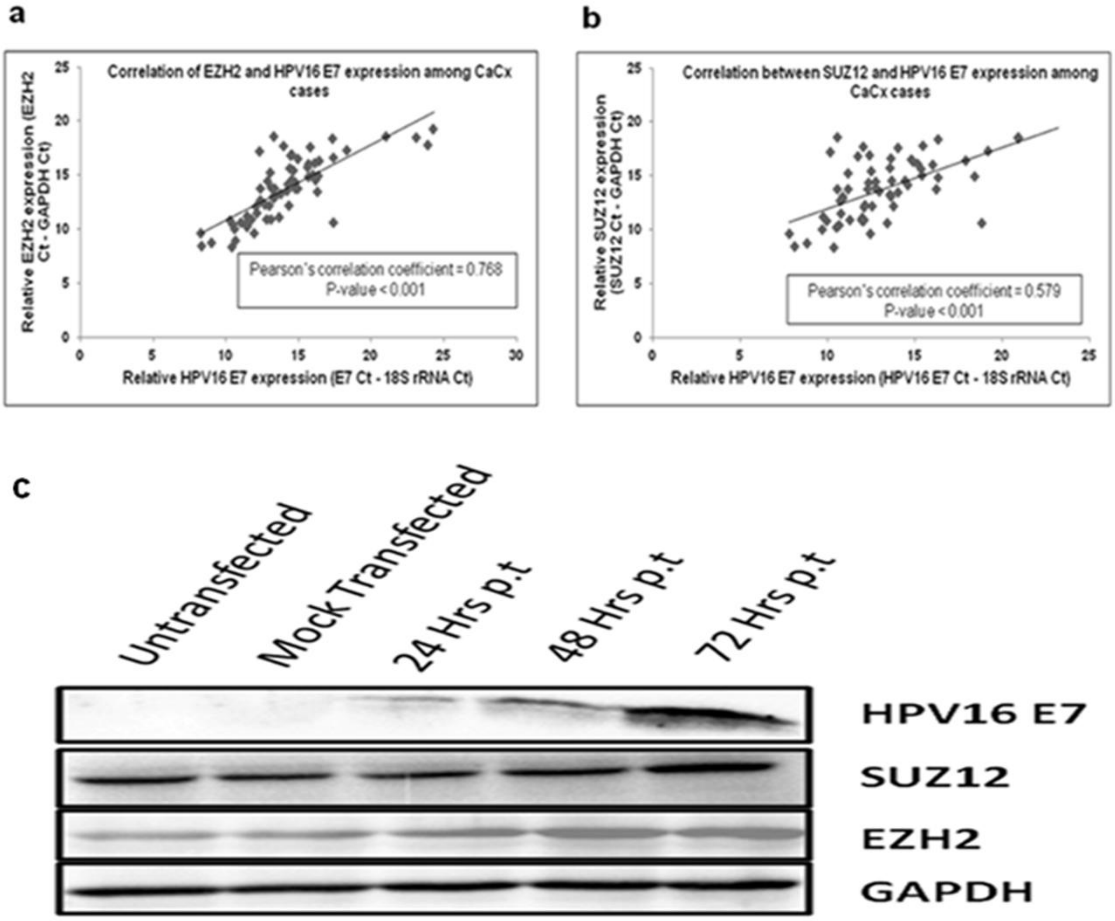

Supplementary Fig. S5| (a). & (b). Correlation analysis of EZH2 and SUZ12 relative expression with HPV16 E7 expression. (c). Immunoblot analysis to identify changes in protein levels of HPV16 E7, EZH2 and SUZ12 - 24, 48 and 72 hrs post transfection of pcDNA3.1-HPV16 E7 as compared to untransfected and mock (empty vector) transfected C33A cells with GAPDH as the loading control. (p. t. – post transfection).

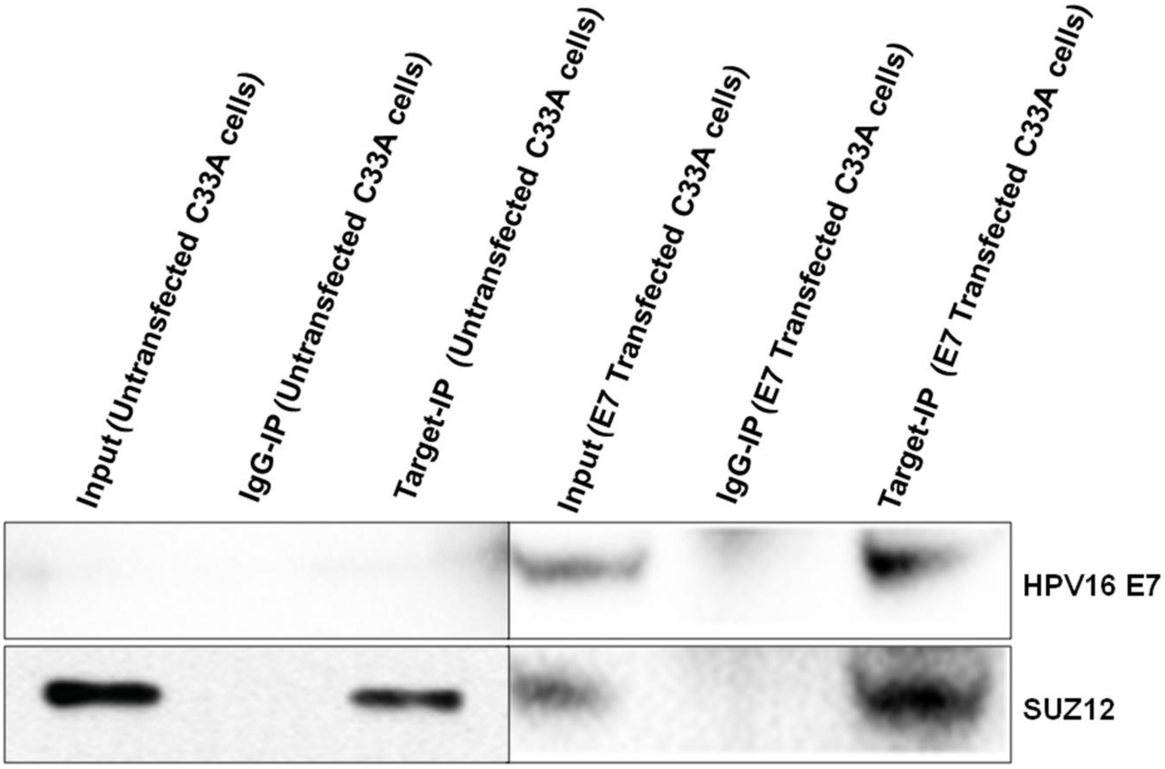

Supplementary Fig. S6| RNA Immunoprecipitation (RIP) Immunoblot for checking specificity of pull-down using Input Cell extract (HPV16 E7/ SUZ12 antibody), IgG, Target protein (HPV16 E7/ SUZ12 antibody) in Untransfected C33A cells and HPV16 E7 expressing C33A cells respectively.

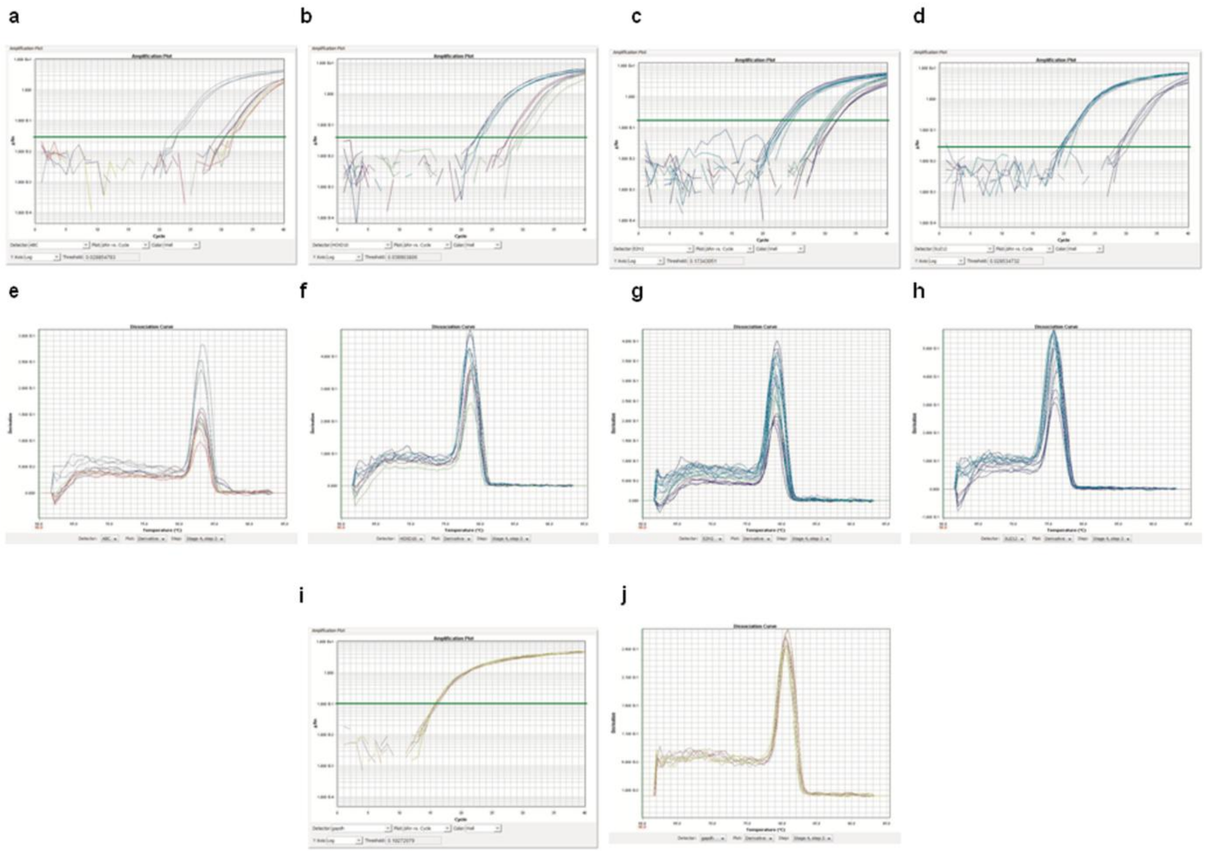

Supplementary Fig. S7| (a).-(d). & (i). Amplification plots of HOTAIR, HOXD10, EZH2, SUZ12 and GAPDH respectively. (e).-(h) & (j). Dissociation curves of HOTAIR, HOXD10, EZH2, SUZ12 and GAPDH respectively.

Supplementary Tables

Supplementary Table S1:

(A) Altered biological processes among low HOTAIR category CaCx cases

Biological Processes	Diseases or Functions Annotation	p-Value	No. of Molecules
Cellular Growth and Proliferation	proliferation of cells	1.28E-15	236
Cell Death and Survival	cell death	1.35E-11	209
Gene Expression	transcription	1.50E-11	129
Gene Expression	transcription of RNA	3.90E-11	126
Cell Death and Survival	apoptosis	1.11E-10	170
Cellular Development, Cellular Growth and Proliferation	proliferation of tumor cell lines	2.50E-10	108
Gene Expression	expression of RNA	2.57E-10	136
Cell Cycle	cell cycle progression	4.00E-10	82
Gene Expression	transcription of DNA	7.99E-09	98
Cancer	breast or colorectal cancer	9.31E-09	244
Gene Expression	expression of DNA	1.56E-08	101
Cell Cycle	arrest in interphase	2.00E-08	40
Cell Cycle	interphase	2.13E-08	55
Cell Death and Survival	necrosis	3.74E-08	158
Cellular Assembly and Organization, Cellular Function and Maintenance, Tissue Development	polymerization of microtubules	2.01E-07	10
DNA Replication, Recombination, and Repair	repair of DNA	2.86E-07	27
Cancer, Organismal Injury and Abnormalities, Reproductive System Disease	mammary tumor	3.41E-07	97
Organismal Survival	organismal death	3.60E-07	144
Cancer, Gastrointestinal Disease	digestive organ	1.86E-	243

	tumor	06	
Cancer	Breast Cancer and Tumors	1.97E-06	89

(B) Altered biological processes among high HOTAIR category CaCx cases

**Biological Processes**	**Diseases or Functions Annotation**	**p-Value**	**No. of Molecules**
Cancer, Organismal Injury and Abnormalities, Reproductive System Disease	uterine serous papillary cancer	6.85E-56	98
Cellular Growth and Proliferation	proliferation of cells	6.44E-39	444
Cellular Movement	cell movement	2.29E-32	284
Cellular Movement	migration of cells	3.77E-32	264
Dermatological Diseases and Conditions	psoriasis	7.44E-32	123
Cell Death and Survival	apoptosis	1.79E-28	328
Cancer	advanced malignant tumor	2.02E-28	137
Cancer	metastasis	7.01E-28	135
Cancer, Organismal Injury and Abnormalities, Reproductive System Disease	genital tumor	4.01E-27	412
Cellular Development, Cellular Growth and Proliferation	proliferation of tumor cell lines	6.99E-27	213
Cancer	Cancer	1.04E-26	866
Cell Death and Survival	cell death	1.29E-26	386
Cell Cycle	cell cycle progression	1.55E-26	165
Cell Cycle	mitosis	6.80E-26	99
Cancer	breast or ovarian cancer	7.23E-26	224
Cancer, Organismal Injury and Abnormalities, Reproductive System Disease	female genital neoplasm	3.06E-25	376
Cancer	pelvic cancer	4.19E-25	392
Cell Death and Survival	necrosis	2.45E-24	312
Cancer	head and neck cancer	1.98E-23	129
Cellular Movement	invasion of cells	4.68E-23	135

(C) Altered biological processes common to low and high HOTAIR category CaCx cases

Biological Processes	Diseases or Functions Annotation	p-Value	No. of Molecules
Cell Cycle	cell cycle progression	2.04E-13	67
Cellular Growth and Proliferation	proliferation of cells	3.97E-13	158
Cell Death and Survival	cell death	5.30E-10	140
Cell Cycle	interphase	5.60E-10	44
Cell Cycle	arrest in interphase	2.39E-09	32
Cell Cycle	mitosis	9.37E-09	34
Cell Death and Survival	apoptosis	1.76E-08	112
Cancer, Organismal Injury and Abnormalities, Reproductive System Disease	mammary tumor	2.35E-08	72
Cancer	breast or colorectal cancer	2.70E-08	164
Cellular Assembly and Organization, Cellular Function and Maintenance, Tissue Development	polymerization of microtubules	4.71E-08	9
Cell Cycle, Cellular Assembly and Organization, DNA Replication, Recombination, and Repair	segregation of chromosomes	8.37E-08	13
Cellular Development, Cellular Growth and Proliferation	proliferation of tumor cell lines	1.51E-07	70
Cancer	breast or ovarian cancer	2.57E-07	74
Cancer, Organismal Injury and Abnormalities, Reproductive System Disease	breast cancer	3.16E-07	65
Cell Cycle	S phase	4.25E-07	21
Gene Expression	expression of RNA	5.63E-07	86
Gene Expression	transcription of RNA	5.68E-07	78
Gene Expression	transcription	5.95E-07	79
Cell Cycle, Reproductive System Development and Function	meiosis of germ cells	7.92E-07	12
DNA Replication, Recombination, and Repair	repair of DNA	1.01E-06	20

(D) Altered biological processes uniquely among low HOTAIR category CaCx cases

**Biological Processes**	**Diseases or Functions Annotation**	**p-Value**	**No. of Molecules**
Gene Expression	transcription	3.78E-06	51
Gene Expression	transcription of RNA	1.11E-05	49
Cell Death and Survival	cell death of kidney cells	7.46E-05	15
Gene Expression	expression of RNA	8.49E-05	51
Gene Expression	expression of DNA	8.95E-05	40
Gene Expression	transcription of DNA	1.30E-04	38
Cellular Assembly and Organization	distribution of early endosomes	1.31E-04	2
Digestive System Development and Function, Embryonic Development, Organ Development, Organismal Development, Tissue Development	formation of intestinal villus	1.31E-04	2
Cell-To-Cell Signaling and Interaction, Hematological System Development and Function, Inflammatory Response	inflammatory response of neutrophils	1.31E-04	2
Cellular Growth and Proliferation	proliferation of cells	1.67E-04	80
Cell Death and Survival	cell death of B-lymphocyte derived cell lines	1.69E-04	8
Cell Death and Survival	cell death of kidney cell lines	1.81E-04	13
Cell Death and Survival	apoptosis of kidney cell lines	2.25E-04	11
Cellular Assembly and Organization	binding of mitochondrial membrane	3.90E-04	2
Cellular Development, Hematological System Development and Function, Hematopoiesis, Humoral Immune Response	differentiation of B lymphocytes	5.04E-04	10
Cellular Development, Cellular Growth and Proliferation	proliferation of tumor cell lines	5.31E-04	38
Cell Morphology	permeabilization of mitochondrial outer membrane	6.13E-04	3
Cell Death and Survival	cell death of cervical cancer cell lines	7.40E-04	13
Developmental Disorder	Growth Failure	7.47E-	20

		04	
Cell Death and Survival	apoptosis of B-lymphocyte derived cell lines	7.64E-04	7

(E) Altered biological processes uniquely among high HOTAIR category CaCx cases

**Biological Processes**	**Diseases or Functions Annotation**	**p-Value**	**No. of Molecules**
Cancer, Organismal Injury and Abnormalities, Reproductive System Disease	uterine serous papillary cancer	6.89E-52	88
Cellular Movement	cell movement	2.80E-33	254
Cellular Movement	migration of cells	9.14E-33	236
Cellular Growth and Proliferation	proliferation of cells	8.75E-32	372
Dermatological Diseases and Conditions	psoriasis	1.27E-30	110
Cancer	advanced malignant tumor	2.03E-27	122
Cancer	metastasis	2.44E-27	121
Cancer, Organismal Injury and Abnormalities, Reproductive System Disease	genital tumor	1.19E-24	353
Cellular Movement	invasion of cells	6.39E-24	123
Cancer	Cancer	2.06E-23	733
Cancer	head and neck cancer	2.25E-23	116
Cancer, Organismal Injury and Abnormalities, Reproductive System Disease	female genital neoplasm	3.54E-23	323
Cellular Development, Cellular Growth and Proliferation	proliferation of tumor cell lines	2.08E-22	179
Cell Death and Survival	apoptosis	4.49E-22	271
Cell Death and Survival	necrosis	7.88E-22	267
Cancer	pelvic cancer	1.28E-21	332
Cancer	head and neck neoplasia	1.45E-21	138
Cancer	benign neoplasia	3.45E-21	108
Cancer	breast or ovarian cancer	1.04E-20	186

Cell Death and Survival	cell death	2.04E-20	319

Supplementary Table S2:

(A) Differentially altered pathways among low HOTAIR category CaCx cases as compared to controls

Ingenuity Canonical Pathways	p-value	Ratio	Molecules	Status
Cell Cycle: G2/M DNA Damage Checkpoint Regulation	1.58489E-05	1.84E-01	CDC25B,CDKN2A,YWHAG,KAT2B,YWHAE,CCNB2,AURKA,BRCA1,CHEK2	Upregulated
Small Cell Lung Cancer Signaling	0.000316228	1.27E-01	PIK3R3,BCL2L1,TRAF2,TRAF3,CCNE1,BID,CDKN1B,NFKBIB,TRAF1	Upregulated
TWEAK Signaling	0.000537032	1.76E-01	TRAF2,TRAF3,BID,NFKBIB,BAG4,TRAF1	Upregulated
Cell Cycle Control of Chromosomal Replication	0.001258925	1.85E-01	MCM3,MCM6,CDC45,CHEK2,MCM7	Upregulated
p53 Signaling	0.003235937	9.18E-02	CDKN2A,PIK3R3,BCL2L1,KAT2B,TOPBP1,TNFRSF10B,PML,BRCA1,CHEK2	Upregulated
Retinol Biosynthesis	0.003630781	1.47E-01	CEL,RDH10,RDH12,RBP1,ESD	Upregulated
CD40 Signaling	0.003715352	1.08E-01	PIK3R3,FOS,TRAF2,TRAF3,STAT3,NFKBIB,TRAF1	Upregulated
Molecular Mechanisms of Cancer	0.004677351	5.75E-02	CDKN2A,BAD,TYK2,RALB,AURKA,CDC25B,PIK3R3,FOS,BCL2L1,CCNE1,RND3,FANCD2,RHOT1,PAK2,BID,RBPJ,CDKN1B,NFKBIB,BRCA1,CHEK2,CAMK2B	Upregulated
PI3K/AKT Signaling	0.004786301	8.13E-02	PIK3R3,BCL2L1,YWHAG,BAD,YWHAE,TYK2,HSP90AA1,INPPL1,CDKN1B,NFKBIB	Upregulated
TCA Cycle II (Eukaryotic)	0.004897788	1.74E-01	SDHA,SUCLA2,DLD,SDHD	Downregulated
UVA-Induced MAPK Signaling	0.005754399	9.09E-02	PLCD1,PIK3R3,BCL2L1,FOS,TIPARP,PARP2,PARP12,PARP14	Upregulated
Role of JAK1, JAK2 and TYK2 in Interferon Signaling	0.005754399	1.67E-01	TYK2,STAT2,STAT3,IFNAR2	Upregulated

April Mediated Signaling	0.005888437	1.32E-01	FOS,TRAF2,TRAF3,NFKBIB,TRAF1	Upregulated
JAK/Stat Signaling	0.006606934	9.72E-02	PIK3R3,BCL2L1,FOS,PIAS4,TYK2,STAT2,STAT3	Upregulated
Glucocorticoid Receptor Signaling	0.00724436	6.13E-02	STAT3,ERCC2,MNAT1,TSG101,TAF7L,SMARCA4,TSC22D3,PIK3R3,FOS,BCL2L1,TRAF2,KAT2B,DUSP1,HSP90AA1,NFKBIB,NOS2	Upregulated
Role of PKR in Interferon Induction and Antiviral Response	0.007413102	1.25E-01	TRAF2,TRAF3,BID,EIF2AK2,NFKBIB	Upregulated
B Cell Activating Factor Signaling	0.007413102	1.25E-01	FOS,TRAF2,TRAF3,NFKBIB,TRAF1	Upregulated
Death Receptor Signaling	0.007413102	8.7E-02	TRAF2,TIPARP,PARP2,TNFRSF10B,BID,PARP12,NFKBIB,PARP14	Upregulated
RAR Activation	0.00851138	6.82E-02	PIK3R3,FOS,KAT2B,RDH10,DUSP1,RDH12,ERCC2,PML,MNAT1,RBP1,CITED2,SMARCA4	Downregulated
Myc Mediated Apoptosis Signaling	0.008709636	1.03E-01	CDKN2A,PIK3R3,YWHAG,BAD,YWHAE,BID	Upregulated

(B) Differentially altered pathways among high HOTAIR category CaCx cases as compared to controls

Ingenuity Canonical Pathways	P-value	Ratio	Molecules	Status
Cell Cycle: G2/M DNA Damage Checkpoint Regulation	2.51189E-11	3.67E-01	CDKN2A,CDC25C,CKS2,CCNB2,PLK1,RPRM,AURKA,CDK1,SKP2,CHEK1,CDC25B,KAT2B,CKS1B,TOP2A,PKMYT1,RPS6KA1,BRCA1,CHEK2	Upregulated
Agranulocyte Adhesion and Diapedesis	5.62341E-09	1.69E-01	IL1A,ICAM1,CXCL12,CCL14,MYH11,CLDN7,CXCR1,CXCL9,CXCL10,CXCL13,CCL8,CCL3L3,CXCL14,CXCL1,MMP11,MMP12,CCL15,MMP1,CXCL8,CCL23,CKLF,MMP10,PODXL2,CLDN8,JAM3,CCL21,IL1B,CCL26,CCL18,CD34,CLDN17,MMP9	Upregulated
Granulocyte Adhesion and Diapedesis	1.62181E-08	1.69E-01	IL1A,ICAM1,CXCL12,CCL14,CLDN7,CXCL9,IL1R2,CXCL10,CXCL13,CCL8,SDC2,CCL3L3,CXCL14,CXCL1,	Upregulated

			MMP11,MMP12,CCL15,MMP1,CXCL8,CCL23,CKLF,MMP10,CLDN8,JAM3,CCL21,IL1B,CCL26,CCL18,CLDN17,MMP9	
Cell Cycle Control of Chromosomal Replication	5.49541E-08	4.07E-01	MCM5,MCM3,MCM6,CDC45,RPA3,ORC6,CHEK2,MCM4,CDK2,MCM7,ORC1	Upregulated
Estrogen-mediated S-phase Entry	1.7378E-07	4.17E-01	CCNA2,CCNE2,CCNE1,E2F3,CCND1,CDK1,E2F2,CDK2,SKP2,CDC25A	Upregulated
Hepatic Fibrosis / Hepatic Stellate Cell Activation	1.90546E-07	1.52E-01	IGFBP4,IL1A,ICAM1,LHX2,FGF2,KLF6,MYH11,CXCL9,COL17A1,VEGFA,IL1R2,TGFBR2,EDN1,IGF1,PDGFRA,STAT1,MMP1,TIMP2,CXCL8,COL4A1,EDNRB,IGFBP5,IFNAR2,CD70,CCL21,EDNRA,IL1B,PDGFD,COL9A2,MMP9	Downregulated
Mitotic Roles of Polo-Like Kinase	2.5704E-07	2.42E-01	KIF23,CDC25C,CDC20,PTTG1,PRC1,CCNB2,PLK1,CDK1,CDC25B,PLK4,PLK2,PKMYT1,FBXO5,KIF11,CHEK2,CDC25A	Upregulated
Aryl Hydrocarbon Receptor Signaling	3.46737E-07	1.71E-01	CDKN2A,GSTM1,IL1A,CCNE2,NFIX,GSTM5,HSPB2,GSTA4,CCND1,CYP1B1,CHEK1,NR2F1,CCNA2,FOS,CCNE1,ALDH1A1,CCND2,ALDH1A2,IL1B,NFIB,CHEK2,CDK2,ALDH7A1,MCM7	Downregulated
GADD45 Signaling	2.81838E-06	4.21E-01	PCNA,CCNE2,CCNE1,CCND2,BRCA1,CCND1,CDK1,CDK2	Upregulated
Role of CHK Proteins in Cell Cycle Checkpoint Control	4.57088E-06	2.36E-01	CDC25C,PLK1,RFC5,E2F3,CDK1,CHEK1,PCNA,RFC4,BRCA1,CHEK2,E2F2,CDK2,CDC25A	Upregulated
Atherosclerosis Signaling	7.4131E-06	1.63E-01	CXCL8,IL1A,ALOX12B,ICAM1,CD36,CXCL12,ALOX12,PLA2G7,ALOXE3,TPSAB1/TPSB2,TNFRSF12A,APOL1,ALOX15B,APOC1,IL1B,PLA2G4F,PDGFD,MMP9,MMP1,APOD	Upregulated
p53 Signaling	1.47911E-05	1.73E-01	CDKN2A,TP53AIP1,JMY,TOPBP1,PIK3R1,TNFRSF10B,RPRM,CCND1,BIRC5,CHEK1,SERPINE2,PCNA,KAT	Upregulated

			2B,CCND2,BRCA1,CHEK2,CDK2	
Role of BRCA1 in DNA Damage Response	1.54882E-05	2.13E-01	FANCG,PLK1,RFC5,E2F3,CHEK1,FANCB,FANCD2,RFC4,STAT1,BRCA1,HLTF,E2F2,CHEK2	Upregulated
ATM Signaling	5.37032E-05	2.03E-01	CDC25C,SMC2,FANCD2,SMC1B,MAPK10,CCNB2,BRCA1,CDK1,CHEK2,CDK2,CDC25A,CHEK1	Upregulated
RAR Activation	6.16595E-05	1.31E-01	RDH10,PIK3R1,SDR16C5,ADCY4,NR2F2,RBP1,NR2F1,VEGFA,FOS,SDR9C7,RBP7,KAT2B,ALDH1A1,DUSP1,ALDH1A2,NCOA1,MAPK10,RDH12,CRABP2,ZBTB16,HLTF,MMP1,CITED2	Downregulated
Mismatch Repair in Eukaryotes	0.000112202	3.75E-01	PCNA,RFC4,FEN1,RFC5,POLD1,EXO1	Upregulated
Fatty Acid α-oxidation	0.000112202	3.75E-01	ALDH2,ALOX12B,ALDH1A1,ALDH1A2,ALOXE3,ALDH7A1	Downregulated
Inhibition of Matrix Metalloproteases	0.000165959	2.31E-01	TIMP3,SDC2,RECK,MMP10,MMP11,MMP12,MMP9,MMP1,TIMP2	Upregulated
Glioma Invasiveness Signaling	0.000177828	1.93E-01	TIMP3,RND3,RHOB,HMMR,PIK3R1,CD44,PLAUR,RHOJ,PLAU,MMP9,TIMP2	Upregulated
Cyclins and Cell Cycle Regulation	0.000223872	1.67E-01	CDKN2A,CCNE2,CCNB2,E2F3,CCND1,CDK1,SKP2,CCNA2,CCNE1,CCND2,E2F2,CDK2,CDC25A	Upregulated

(C) Differentially altered pathways common to high HOTAIR category CaCx cases and low HOTAIR category CaCx cases

**Ingenuity Canonical Pathways**	**p-value**	**Ratio**	**Molecules**	**Status**
Cell Cycle: G2/M DNA Damage Checkpoint Regulation	4.365E-06	1.63E-01	CDC25B,CDKN2A,KAT2B,YWHAE,CCNB2,AURKA,BRCA1,CHEK2	Upregulated
Cell Cycle Control of Chromosomal Replication	0.0001549	1.85E-01	MCM3,MCM6,CDC45,CHEK2,MCM7	Upregulated
RAR Activation	0.000182	6.82E-02	PIK3R3,FOS,KAT2B,RDH10,DUSP1,RDH12,ERCC2,P	Downregulation

			ML,MNAT1,RBP1,CITED2,SMARCA4	
Small Cell Lung Cancer Signaling	0.0004677	9.86E-02	PIK3R3,TRAF2,TRAF3,CCNE1,BID,NFKBIB,TRAF1	Upregulated
TWEAK Signaling	0.0004786	1.47E-01	TRAF2,TRAF3,BID,NFKBIB,TRAF1	Upregulated
Retinol Biosynthesis	0.0004786	1.47E-01	CEL,RDH10,RDH12,RBP1,ESD	Upregulated
Glucocorticoid Receptor Signaling	0.0006457	5.36E-02	ERCC2,MNAT1,TSG101,TAF7L,SMARCA4,TSC22D3,PIK3R3,FOS,TRAF2,KAT2B,DUSP1,HSP90AA1,NOS2,NFKBIB	Upregulated
p53 Signaling	0.0006607	8.16E-02	CDKN2A,PIK3R3,KAT2B,TOPBP1,TNFRSF10B,PML,BRCA1,CHEK2	Upregulated
April Mediated Signaling	0.0008128	1.32E-01	FOS,TRAF2,TRAF3,NFKBIB,TRAF1	Upregulated
ATM Signaling	0.001	1.02E-01	FANCD2,CCNB2,BID,CBX5,BRCA1,CHEK2	Upregulated
Role of PKR in Interferon Induction and Antiviral Response	0.0010233	1.25E-01	TRAF2,TRAF3,BID,EIF2AK2,NFKBIB	Upregulated
B Cell Activating Factor Signaling	0.0010233	1.25E-01	FOS,TRAF2,TRAF3,NFKBIB,TRAF1	Upregulated
Role of BRCA1 in DNA Damage Response	0.0012023	9.84E-02	FANCB,FANCD2,RFC4,BRCA1,CHEK2,SMARCA4	Upregulated
CD40 Signaling	0.0016596	9.23E-02	PIK3R3,FOS,TRAF2,TRAF3,NFKBIB,TRAF1	Upregulated
Hereditary Breast Cancer Signaling	0.0018621	6.96E-02	UBD,PIK3R3,FANCB,FANCD2,RFC4,BRCA1,CHEK2,SMARCA4	Upregulated
Death Receptor Signaling	0.002138	7.61E-02	TRAF2,PARP2,TNFRSF10B,BID,PARP12,NFKBIB,PARP14	Upregulated
TNFR2 Signaling	0.0022909	1.38E-01	FOS,TRAF2,NFKBIB,TRAF1	Upregulated
The Visual Cycle	0.0028184	2E-01	RDH10,RDH12,RBP1	Upregulated
Mismatch Repair in Eukaryotes	0.0033884	1.88E-01	RFC4,FEN1,POLD1	Upregulated
DNA damage-induced 14-3-3σ Signaling	0.0056234	1.58E-01	CCNE1,CCNB2,BRCA1	Upregulated

(D) Differentially altered pathways uniquely among low HOTAIR category CaCx cases as compared to controls

Ingenuity Canonical Pathways	p-value	Ratio	Molecules	Status
Acetyl-CoA Biosynthesis I (Pyruvate Dehydrogenase Complex)	0.0026303	2.86E-01	DLAT,DLD	Downregulated
GM-CSF Signaling	0.0056234	6.45E-02	BCL2L1,PIM1,STAT3,CAMK2B	Upregulated
ERK/MAPK Signaling	0.0058884	3.74E-02	YWHAG,PPP1R10,BAD,PPP1R7,PAK2,STAT3,ELF1	Downregulated
DNA Methylation and Transcriptional Repression Signaling	0.0218776	1E-01	MBD3,ARID4B	Upregulated
ErbB2-ErbB3 Signaling	0.0275423	5.26E-02	BAD,STAT3,CDKN1B	Downregulated
TCA Cycle II (Eukaryotic)	0.0281838	8.7E-02	SUCLA2,DLD	Downregulated
Rac Signaling	0.0316228	3.85E-02	PAK2,ARPC4,NCKAP1,ANK1	Upregulated
Pancreatic Adenocarcinoma Signaling	0.0338844	3.77E-02	BCL2L1,BAD,STAT3,CDKN1B	Downregulated
Methylglyoxal Degradation I	0.0338844	3.33E-01	GLO1	Downregulated
Thyroid Hormone Biosynthesis	0.0338844	3.33E-01	CTSD	Downregulated
Oxidized GTP and dGTP Detoxification	0.0338844	3.33E-01	DDX6	Upregulated
2-ketoglutarate Dehydrogenase Complex	0.0446684	2.5E-01	DLD	Downregulated
Branched-chain α-keto acid Dehydrogenase Complex	0.0446684	2.5E-01	DLD	Downregulated
Myo-inositol Biosynthesis	0.0446684	2.5E-01	ISYNA1	Upregulated
14-3-3-mediated Signaling	0.0457088	3.42E-02	YWHAG,BAD,CDKN1B,PDCD6IP	Downregulated
PI3K/AKT Signaling	0.0537032	3.25E-02	BCL2L1,YWHAG,BAD,CDKN1B	Downregulated
Ceramide Biosynthesis	0.0562341	2E-01	SPTLC1	Downregulated
2-oxobutanoate Degradation I	0.0562341	2E-01	DLD	Downregulated
Acute Myeloid Leukemia Signaling	0.0588844	3.9E-02	BAD,PIM1,STAT3	Downregulated
Molecular Mechanisms of Cancer	0.060256	2.19E-02	BCL2L1,BAD,RHOT1,RALB,PAK2,RBPJ,CDKN1B,CA	Upregulated

			MK2B	

(E) Differentially altered pathways uniquely among High HOTAIR category CaCx cases as compared to controls

Ingenuity Canonical Pathways	P-value	Ratio	Molecules	Status
Cell Cycle: G2/M DNA Damage Checkpoint Regulation	2.51189E-11	3.67E-01	CDKN2A,CDC25C,CKS2,CCNB2,PLK1,RPRM,AURKA,CDK1,SKP2,CHEK1,CDC25B,KAT2B,CKS1B,TOP2A,PKMYT1,RPS6KA1,BRCA1,CHEK2	Upregulated
Agranulocyte Adhesion and Diapedesis	5.62341E-09	1.69E-01	IL1A,ICAM1,CXCL12,CCL14,MYH11,CLDN7,CXCR1,CXCL9,CXCL10,CXCL13,CCL8,CCL3L3,CXCL14,CXCL1,MMP11,MMP12,CCL15,MMP1,CXCL8,CCL23,CKLF,MMP10,PODXL2,CLDN8,JAM3,CCL21,IL1B,CCL26,CCL18,CD34,CLDN17,MMP9	Upregulated
Granulocyte Adhesion and Diapedesis	1.62181E-08	1.69E-01	IL1A,ICAM1,CXCL12,CCL14,CLDN7,CXCL9,IL1R2,CXCL10,CXCL13,CCL8,SDC2,CCL3L3,CXCL14,CXCL1,MMP11,MMP12,CCL15,MMP1,CXCL8,CCL23,CKLF,MMP10,CLDN8,JAM3,CCL21,IL1B,CCL26,CCL18,CLDN17,MMP9	Upregulated
Cell Cycle Control of Chromosomal Replication	5.49541E-08	4.07E-01	MCM5,MCM3,MCM6,CDC45,RPA3,ORC6,CHEK2,MCM4,CDK2,MCM7,ORC1	Upregulated
Estrogen-mediated S-phase Entry	1.7378E-07	4.17E-01	CCNA2,CCNE2,CCNE1,E2F3,CCND1,CDK1,E2F2,CDK2,SKP2,CDC25A	Upregulated
Hepatic Fibrosis / Hepatic Stellate Cell Activation	1.90546E-07	1.52E-01	IGFBP4,IL1A,ICAM1,LHX2,FGF2,KLF6,MYH11,CXCL9,COL17A1,VEGFA,IL1R2,TGFBR2,EDN1,IGF1,PDGFRA,STAT1,MMP1,TIMP2,CXCL8,COL4A1,EDNRB,IGFBP5,IFNAR2,CD70,CCL21,EDNRA,IL1B,PDGFD,COL9A2,MMP9	Downregulated
Mitotic Roles of Polo-Like Kinase	2.5704E-07	2.42E-01	KIF23,CDC25C,CDC20,PTTG1,PRC1,CCNB2,PLK1,CD	Upregulated

			K1,CDC25B,PLK4,PLK2,PKMYT1,FBXO5,KIF11,CHEK2,CDC25A	
Aryl Hydrocarbon Receptor Signaling	3.46737E-07	1.71E-01	CDKN2A,GSTM1,IL1A,CCNE2,NFIX,GSTM5,HSPB2,GSTA4,CCND1,CYP1B1,CHEK1,NR2F1,CCNA2,FOS,CCNE1,ALDH1A1,CCND2,ALDH1A2,IL1B,NFIB,CHEK2,CDK2,ALDH7A1,MCM7	Downregulated
GADD45 Signaling	2.81838E-06	4.21E-01	PCNA,CCNE2,CCNE1,CCND2,BRCA1,CCND1,CDK1,CDK2	Upregulated
Role of CHK Proteins in Cell Cycle Checkpoint Control	4.57088E-06	2.36E-01	CDC25C,PLK1,RFC5,E2F3,CDK1,CHEK1,PCNA,RFC4,BRCA1,CHEK2,E2F2,CDK2,CDC25A	Upregulated
Atherosclerosis Signaling	7.4131E-06	1.63E-01	CXCL8,IL1A,ALOX12B,ICAM1,CD36,CXCL12,ALOX12,PLA2G7,ALOXE3,TPSAB1/TPSB2,TNFRSF12A,APOL1,ALOX15B,APOC1,IL1B,PLA2G4F,PDGFD,MMP9,MMP1,APOD	Upregulated
p53 Signaling	1.47911E-05	1.73E-01	CDKN2A,TP53AIP1,JMY,TOPBP1,PIK3R1,TNFRSF10B,RPRM,CCND1,BIRC5,CHEK1,SERPINE2,PCNA,KAT2B,CCND2,BRCA1,CHEK2,CDK2	Upregulated
Role of BRCA1 in DNA Damage Response	1.54882E-05	2.13E-01	FANCG,PLK1,RFC5,E2F3,CHEK1,FANCB,FANCD2,RFC4,STAT1,BRCA1,HLTF,E2F2,CHEK2	Upregulated
ATM Signaling	5.37032E-05	2.03E-01	CDC25C,SMC2,FANCD2,SMC1B,MAPK10,CCNB2,BRCA1,CDK1,CHEK2,CDK2,CDC25A,CHEK1	Upregulated
RAR Activation	6.16595E-05	1.31E-01	RDH10,PIK3R1,SDR16C5,ADCY4,NR2F2,RBP1,NR2F1,VEGFA,FOS,SDR9C7,RBP7,KAT2B,ALDH1A1,DUSP1,ALDH1A2,NCOA1,MAPK10,RDH12,CRABP2,ZBTB16,HLTF,MMP1,CITED2	Downregulated
Mismatch Repair in Eukaryotes	0.000112202	3.75E-01	PCNA,RFC4,FEN1,RFC5,POLD1,EXO1	Upregulated
Fatty Acid α-oxidation	0.000112202	3.75E-01	ALDH2,ALOX12B,ALDH1A1,ALDH1A2,ALOXE3,ALDH7A1	Downregulated

Inhibition of Matrix Metalloproteases	0.000165959	2.31E-01	TIMP3,SDC2,RECK,MMP10,MMP11,MMP12,MMP9,MMP1,TIMP2	Upregulated
Glioma Invasiveness Signaling	0.000177828	1.93E-01	TIMP3,RND3,RHOB,HMMR,PIK3R1,CD44,PLAUR,RHOJ,PLAU,MMP9,TIMP2	Upregulated
Cyclins and Cell Cycle Regulation	0.000223872	1.67E-01	CDKN2A,CCNE2,CCNB2,E2F3,CCND1,CDK1,SKP2,CCNA2,CCNE1,CCND2,E2F2,CDK2,CDC25A	Upregulated
